# A Gap Junction Protein, Inx2, Modulates Calcium Flux to Specify Border Cell Fate during *Drosophila* oogenesis

**DOI:** 10.1371/journal.pgen.1006542

**Published:** 2017-01-23

**Authors:** Aresh Sahu, Ritabrata Ghosh, Girish Deshpande, Mohit Prasad

**Affiliations:** 1 Department of Biological Sciences Indian Institute of Science Education & Research Kolkata Mohanpur Campus Mohanpur, Nadia, West Bengal, India; 2 Department of Molecular Biology Princeton University, Princeton, NJ, United States of America; 3 Indian Institute of Science Education and Research Pune. Pune Maharashtra, India; The University of North Carolina at Chapel Hill, UNITED STATES

## Abstract

Intercellular communication mediated by gap junction (GJ) proteins is indispensable during embryogenesis, tissue regeneration and wound healing. Here we report functional analysis of a gap junction protein, Innexin 2 (Inx2), in cell type specification during *Drosophila* oogenesis. Our data reveal a novel involvement of Inx2 in the specification of Border Cells (BCs), a migratory cell type, whose identity is determined by the cell autonomous STAT activity. We show that Inx2 influences BC fate specification by modulating STAT activity via Domeless receptor endocytosis. Furthermore, detailed experimental analysis has uncovered that Inx2 also regulates a calcium flux that transmits across the follicle cells. We propose that Inx2 mediated calcium flux in the follicle cells stimulates endocytosis by altering Dynamin (Shibire) distribution which is in turn critical for careful calibration of STAT activation and, thus for BC specification. Together our data provide unprecedented molecular insights into how gap junction proteins can regulate cell-type specification.

## Author Summary

Gap junction mediated intercellular communication modulates several processes during development, morphogenesis and normal tissue homeostasis. While gap junction proteins play an important role during intercellular communication, the underlying molecular mechanism(s) as to how they regulate diverse signaling cascades are unclear. By employing the *Drosophila melanogaster* oogenesis model we have characterized the role of gap junction protein, Innexin 2 (Inx2), in cell fate specification during *Drosophila* oogenesis. Our data demonstrate that loss of *inx2* affects border cell specification. Border cells are a small group of 6–8 follicle cells that acquire migratory fate in response to the activation of JAK-STAT signaling. We show that perturbing Inx2 levels in the follicle cells inhibits JAK-STAT signaling thereby adversely influencing border cell fate specification. Using live cell imaging and molecular genetic analysis, we have elucidated the molecular mechanism underlying Inx2 function in this process. We show that Inx2 mediates inter-follicular calcium flux that is critical for border cell fate determination. Furthermore, our observations indicate that Inx2 regulates Domeless receptor internalization possibly via influencing distribution of *Drosophila* Dynamin, Shibire in the follicle cells. Taken together these results suggest a functional link between Inx2, calcium flux and receptor endocytosis during border cell fate specification in *Drosophila* oogenesis.

## Introduction

Multicellular development in higher eukaryotes is critically dependent upon proper cell fate specification. Molecular mechanisms underlying cell fate specification fall into two broad categories. Cell autonomous mode of fate specification depends upon intrinsic factors that typically comprise of transcriptional regulators whereas non-autonomous mechanisms are initiated by either paracrine or juxtacrine signaling between different cell types. Typically a combination of cell autonomous and non-autonomous mechanisms is employed to achieve proper specification of different cell types. Communication between different cell types is thus critical to determine the molecular nature of the combinatorial code that ultimately decides the cell type identity.

Proper specification of distinct cell types is hence thought to be a combined outcome of canonical mechanisms that rely upon the cell autonomous transcriptional regulators and ligand- receptor interactions between neighboring and/or surrounding cells. In addition, recent data have suggested that intercellular communication mediated by the gap junctions may also modulate cell fate determination in multicellular systems including *C*. *elegans*, mammals and planaria [1–4].

Oogenesis in *Drosophila melanogaster* has emerged as an attractive model system to elucidate diverse mechanisms underlying cell fate specification in large part due to the heterogeneous cell types present in an adult ovary. A *Drosophila* ovary consists of both somatic and germ line cells that are packaged into oval shaped structures called the egg chambers. Within an egg chamber, a layer of somatic follicle epithelial cells surrounds 16 centrally located germ line cells. As the oogenesis proceeds, the posterior germ line cell acquires oocyte fate while the remaining 15, serve as the nurse cells that support oocyte growth and development.

By contrast, the follicle cells which are epithelial in nature, comprise of three main subgroups; the stalk cells, polar cells and the main body follicle cells [5]. Among these three subtypes, the polar cells mark the poles of an egg chamber and aid in the specification of migratory group of cells, termed as border cells (BCs) from the anterior follicle cells. Specification of BCs is of special interest since this fate transformation involves partial epithelial to mesenchymal transition reminiscent of various developmental and pathological events including morphogenesis and tumor metastasis [6].

The polar cells secrete diffusible cytokine ligand, Unpaired (Upd), which activates the JAK-STAT pathway in the neighboring follicle cells. STAT activation transforms 6–8 anterior follicle cells to acquire border cell fate [7]. Because of the intriguing phenotypic traits including subsequent migratory behavior of the BC population, the modulation of the JAK-STAT pathway in this context is under constant scrutiny. In this regard it is noteworthy that among the 8 gap junction proteins from the *Drosophila* genome {Ogre, Innexin (inx) 2–7}, several exhibit a distinct spatial pattern of expression in somatic and germ line cells of the developing ovary [8]. In particular, *inx2* transcripts have been detected in the anterior end of the early stage egg chamber and appear to overlap with the follicle cells that eventually give rise to the BCs [8]. We therefore decided to investigate possible functions of some of these gap junction proteins during acquisition of distinct cellular identities including BC fate during oogenesis in *Drosophila melanogaster*.

The highly conserved gap junction proteins have been implicated in controlling diverse functions such as embryonic patterning, morphogenesis and tissue homeostasis from sea urchins to higher mammals [9,10]. In general, the functional gap junction channel is formed by the head to head alignment of two hemichannels contributed by each participating cell. Hemichannels are hexameric complexes that are formed by the oligomerization of four pass trans-membrane gap junction proteins, which allow for a controlled passage of small molecules/ions [9]. Consistent with this special structural configuration, the gap junctions have acquired versatile functions. For instance, some members of the gap junction channel family are involved in regulation of excitable cell populations such as neurons, which generate electrically coupled rapid and synchronized responses [11]. On the other hand, different members of the same family help execute efficient protein trafficking to facilitate metabolic coupling [11,12]. To document the involvement of gap junction proteins, these studies have employed either traditional ‘loss-of-function’ mutant analysis or application of small inhibitory molecules. Consequently, while the contribution of the respective gap junction proteins during cell type specification is apparent, our understanding of the underlying molecular mechanisms is far from clear.

In this study, we report a novel function of a gap junction protein, Inx2, during specification of BC fate. Our data demonstrate that Inx2 achieves this by modulating the JAK-STAT signaling in the follicle cells. Furthermore, we posit a three-component regulatory module between Inx2, Dynamin and calcium transport that influences the inter-follicular communication ultimately responsible for the determination of BC fate.

## Results

### Innexin2 is required for BC specification during oogenesis

**Fig 1:**
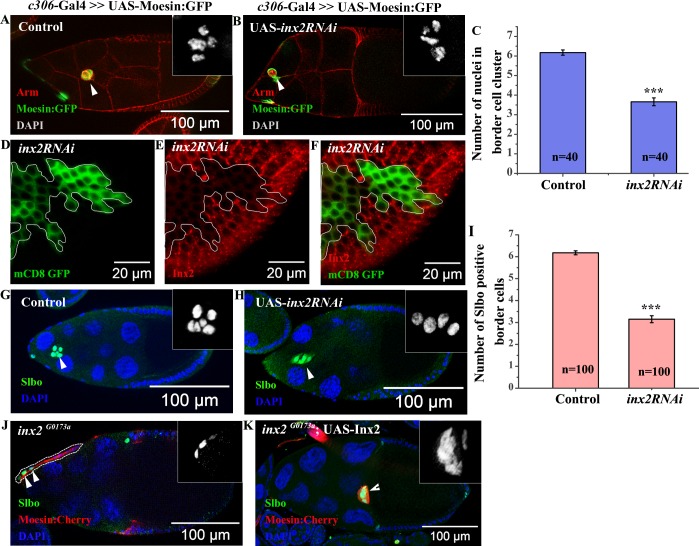
Inx2 is required for border cell (BC) fate specification. (A, B, G, H): Stage 9–10 egg chambers of indicated genotypes. Arrowheads mark the cluster. (A, B): anti-Armadillo (Red), GFP (Green). Inset shows magnified image of BC nuclei stained with DAPI. (C): Histogram compares the number of BC nuclei of the genotype represented in (A, B). (D-F): Mosaic analysis employing Actin<Flipout >Gal4 resulting in overexpression of *inx2RNAi*. Follicle cell overexpressing clones are outlined in white. GFP (Green) (D) and anti-Inx2 (Red) (E). (F): Merge of (D) and (E). Note the down regulation in levels of Inx2 protein in clones over expressing *inx2RNAi* construct compared to nearby wild type cells. (G, H): anti-Slbo antibody (Green) and DAPI (Blue). Inset represents magnified image of Slbo positive BC. (I): Corresponding histogram representing number of BCs in (G) and (H). Note the decrease in the number of BCs in Inx2-depleted clusters. (J, K): MARCM analysis. Clones are identified by Moesin:Cherry (Red) expression. Arrowheads mark BCs stained with anti-Slbo (Green). (J): Dotted line marks *inx2* mutant cells. Inset is single channel magnified image of the anterior end of the sample shown in (J). Note the absence of BC cluster. (K): Single plane image of stage 10 egg chamber of *inx2*^*G0173a*^ homozygous MARCM mutant clones co-expressing Inx2cDNA. The rescue of the Inx2-depleted phenotype is evident with conspicuous BC cluster formation and their efficient migration. Inset represents magnified image of Slbo positive border cells (Slbo). ‘n’ represents number of egg chambers analyzed. Error bar represents Standard Error of Mean. *** represents p-value <0.001.

The gap junction genes *inx2*, *inx3*, *inx7* and *ogre* are known to be expressed in the follicle cells and the migratory cells of the *Drosophila* egg chamber [8,13]. Among this group, the *inx2* transcripts are detected specifically in the anterior follicle cells of early stage egg chambers [8]. Since a subset of 6–8 anterior follicle cells acquire border cell fate, we were curious if the early and localized expression of *inx2*, has any role in establishing the BC identity during *Drosophila* oogenesis [8]. We initially tested this idea by over expression of *inx2RNAi* using *c306*-Gal4 driver that shows robust expression in the anterior follicle cells of the *Drosophila* egg chambers [14]. Interestingly, down regulation of Inx2 function in the anterior follicle cells resulted in smaller BC clusters with lesser number of cells in the migrating cohort as opposed to wild type. Control BC clusters have around 6.2±0.1 nuclei (area 714±27μm^2^) whereas the Inx2-depleted clusters consist of 3.7±0.2 nuclei (area 468±28 μm^2^) (Fig 1A–1C and S1A Fig). For optimum expression, all the experiments involving *inx2RNAi* and the corresponding controls were carried out at 29°c unless otherwise stated. Next we sought to test if the *inx2RNAi* construct indeed targets Inx2. We immunostained egg chambers with Inx2 antibody where random flipout clones over expressing the *inx2RNAi* construct were generated. Consistent with our expectation, follicle cells over expressing *inx2RNAi* construct indeed exhibited lower levels of Inx2 protein compared to their wild type counterparts (Fig 1D–1F). To ascertain the specificity of the *inx2RNAi* construct further, we performed a rescue experiment by expressing Inx2cDNA in the Inx2-depleted follicle cells. On an average the Inx2-depleted border cell clusters exhibit around 3.5 nuclei per cluster (average 3.5±0.4). Since BC clusters coexpressing Inx2cDNA and *inx2RNAi* were larger with higher number of nuclei per cluster (average 5.8±0.2), it suggested that increasing Inx2 levels rescues border cell fate in Inx2-depleted follicle cells (S1B–S1F Fig). In addition, we also observed rescue of migratory fate when we over expressed C terminal tagged Inx2:RFP construct that is known to mimic the wild type protein, in the Inx2-depleted clusters [15] (S1E Fig). Altogether our data validated the specificity of the *inx2RNAi* construct and suggested that phenotypes associated with *inx2RNAi* over expression are indeed due to down regulation of Inx2 activity.

Since the average number of nuclei in Inx2-depleted clusters is consistently lower than the control, we were curious if the early specification and recruitment of cells to the BC fate was compromised. Alternatively, BCs could fall off the moving cluster due to poor adhesion thus resulting in smaller cohorts. To distinguish between the two possibilities, we stained the egg chambers with an antibody specific for a C/EBP transcription factor, Slow Border Cell (Slbo). Slbo is activated by the JAK-STAT signaling in a subset of anterior follicle cells, which eventually acquires BC fate. Consistent with our earlier observations, we found considerably reduced number of Slbo positive cells (average 3.2±0.16) in the Inx2-depleted BC cluster compared to the control (average 6.2±0.1) (Fig 1G–1I). Interestingly, although smaller in size, mutant clusters were always intact as in the case of wild type. Moreover, we did not observe any lagging Slbo positive cell/s fallen off from the migrating cluster. These observations suggested that small size of Inx2-depleted cluster was possibly due to inefficient recruitment of the follicle cells to the BC fate.

Next we wondered if Inx2 protein functions as a channel during BC specification. To examine this possibility we decided to employ RFP tagged Inx2 (RFP:Inx2) construct that has been shown to specifically compromise channel activity of Inx2 [15]. As in the case of *inx2RNAi*, over expression of RFP tagged Inx2 (RFP:Inx2) construct also resulted in specification of fewer border cells (5.04±0.18) in the migrating cluster as compared to control (6.6±0.07) [15] (S1G–S1I Fig). This observation suggested that Inx2 likely functions as a component of a channel during the specification of border cells.

Border cell specification defects usually lead to altered migration efficiency of the cluster. Since the Inx2-depleted clusters are smaller we wondered if their migration efficiency is also compromised. Indeed Inx2-depleted clusters exhibited migration defect as only 38% of the clusters reached the oocyte boundary unlike 97% observed in the wild type control samples **(**S1J–S1L Fig**)**. This observation further supported our hypothesis that Inx2 modulates border cell fate specification during *Drosophila* oogenesis.

To confirm the RNAi dependent phenotypes, we used two different *inx2* alleles reported in the flybase. Mosaic analysis with *inx2*^*G0059*^ (DGRC 114609) and *inx2*^*G0173a*^ (DGRC 111858) recapitulated the phenotypes induced by *inx2RNAi*. Anterior follicle cells homozygous for *inx2*^*G0173a*^ exhibited substantially reduced number of BCs, with complete absence of the cluster in 45% of the egg chambers (n = 22) (Fig 1J). Similar observation was made when *inx2*^*G0059*^ (DGRC 114609) allele was employed. As in the case of *inx2*^*G0173a*^ allele, 28% of the egg chambers with *inx2*^*G0059*^ mutant anterior follicle cells completely lacked the border cell cluster (n = 29). In addition, both the *inx2*^*G0173a*^ (n = 22) and *inx2*^*G0059*^ (n = 29) mutant egg chambers displayed migration defects in 82% and 76% of stage 10 egg chambers respectively. Over expression of the Inx2cDNA using *Actin*-Gal4 rescued border cell fate resulting in the appearance of near wild-type BC cluster in all the *inx2*^*G0173a*^ mutant egg chambers. (n = 39). Moreover the frequency of migration defects was also reduced significantly from 82% to 39% (n = 39). Taken together these data suggest that *inx2*^*G0173a*^ is indeed a bonafide allele of *inx2* (Fig 1K). Altogether these data support the conclusion that consistent with its expression in the anterior follicle cells, *inx2* plays a crucial role during BC fate specification and, probably as a consequence, also influences their migratory behaviour.

Within the anterior follicle cells, the interaction between the polar cells and its adjacent follicle cells is critical for the specification of BCs [7]. We were thus curious if Inx2 functions in the polar cells to modulate the border cell fate. To test this we downregulated Inx2 function by over expressing *inx2RNAi* using polar cell specific driver *Upd*-Gal4. To confirm that the Inx2 levels were indeed affected in Inx2-depleted polar cells, we first stained the egg chambers with anti-Inx2 antibody. At higher magnification, we observed punctate staining at the interface of the two polar cells and this was reduced in Inx2-depleted background (S2A’ and S2B’ Fig). Quantification of the egg chambers indicated that compared to the control (0%), 63% of the Inx2-depleted polar cells exhibit reduction in Inx2 levels (number of egg chambers analyzed = 11).

Despite the reduction in the protein levels, overexpression of *inx2RNAi* by *Upd*-Gal4 didn’t affect the border cell fate or border cell migration appreciably. (5.9±0.6 nuclei compared to control of 6.2±0.7 nuclei) (S2C–S2E Fig). This observation suggested that Inx2 likely functions in the follicle cells adjacent to the polar cells that eventually acquire the border cell identity (S2C–S2E Fig).

Next we were curious to know how Inx2 modulates border cell fate in the follicle cells.

### Inx2 affects JAK-STAT signaling

**Fig 2:**
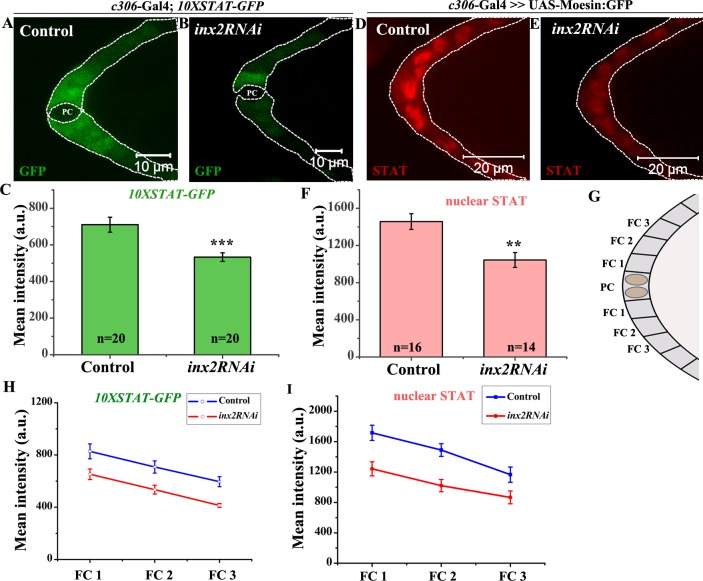
Loss of *inx-2* leads to compromised STAT signaling and STAT levels in the follicle cells. (A, B, D, E): White dotted line outlines anterior follicle cells of Stage 8 egg chamber of indicated genotypes. (A-B): *10XSTAT-GFP* expression. PC denotes polar cells. (C): Histogram compares *10XSTAT-GFP* levels for genotypes represented in (A) and (B). (D, E): STAT expression (Red). (F): Histogram comparing nuclear STAT level for genotypes represented in (D) and (E). Note the decrease in the levels of both *10XSTAT-GFP* and STAT protein in Inx2-depleted follicle cells. (G): Schematic of anterior end of stage 8 egg chamber. FC stands for follicle cell. (H-I): Graph represents *10XSTAT-GFP* (H) and nuclear STAT (I) level in FC1, FC2 and FC3 between control and *inx2RNAi*. Error bar represents Standard Error of Mean. ‘n’ indicates number of egg chambers analyzed. ** p-value <0.01.*** p-value <0.001.

Activation of the JAK-STAT pathway in the follicle cells is critical for the specification of BCs. Cell autonomous STAT activity distinguishes the migrating population i.e. (BCs) from the anterior follicle cells [7]. Since the total number of cells in the Inx2 compromised clusters was significantly reduced compared to wild type, it was conceivable that JAK-STAT signaling is compromised in Inx2-depleted follicle cells. To assess this possibility, we examined *10XSTAT-GFP* reporter which has been routinely used for evaluating the level of JAK-STAT signaling in various *Drosophila* tissues [16]. In the case of this reporter, the intensity of GFP is directly proportional to the degree of activation of JAK-STAT pathway. In the wild type BCs we observe average reporter activity of 1644±157 a.u. while in Inx2-depleted clusters, it was lower with an average activity of around 727±32a.u. (S3A–S3G Fig). To confirm that *10XSTAT-GFP* reporter is downregulated prior to BC specification, we evaluated it’s activity in the younger egg chambers (Stage 8). Similar to the late stage egg chambers with intact clusters, the intensity of the STAT reporter in stage 8 egg chambers in the anterior follicle cells was also diminished in the Inx2-depleted follicle cells. The average intensity of reporter activity in the control follicle cells was 710.4±40.7 a.u. while that of Inx2-depleted follicle cells was observed around 533.5±23.4 a.u. (Fig 2A–2C). In wild type egg chambers, a gradient of STAT reporter activity is observed in the anterior follicle cells [17]. We thus wondered whether the establishment and/or maintenance of the gradient is disrupted in the egg chambers compromised for the Inx2 activity. To examine this possibility we plotted GFP intensity from *10XSTAT-GFP* reporter as a function of the distance of the follicle cells from the polar cells. As reported previously, in the wild type egg chambers, we observe highest level of GFP in the cells adjacent to the polar cells (FC1), intermediate levels for next cells (FC2) and least for cells (FC3) that are farthest [17] (Fig 2C, 2G and 2H). Though we observed a similar STAT reporter gradient in the Inx2-depleted follicle cells, the overall GFP intensity was much lower compared to control. Lower level of STAT is known to activate Apontic (Apt), which in turn, represses expression of both *slbo* and *STAT* thus interfering with border cell specification [17]. These data suggest that downregulation of Inx2 function attenuates JAK-STAT signaling, which is important for BC fate specification during *Drosophila* oogenesis.

Since *10XSTAT-GFP* reporter is activated by the STAT transcription factor, we also examined the levels of STAT protein in Inx2-depleted follicle cells [18]. As reported previously, in the wild type follicle cells, we observed a very distinct nuclear enrichment of STAT protein with a readily detectable gradient from the anterior to posterior [17]. The highest STAT protein was observed at the anterior tip which progressively reduced as the distance from the polar cell increased. By contrast, in the Inx2-depleted follicle cells, the nuclear enrichment of STAT protein was weak and in some instances quite diffuse (Fig 2D–2E). Though the quantification of the nuclear STAT levels indicated presence of a gradient, analogous to the *10XSTAT-GFP* reporter, the staining intensity was lower (Fig 2F, 2G and 2I). Taken together these data suggest that Inx2 regulates STAT levels (and activity) to modulate JAK-STAT signaling in the follicle cells during BC fate specification.

### BC fate specification defects induced due to loss of Inx2 can be alleviated by overexpressing STAT

**Fig 3:**
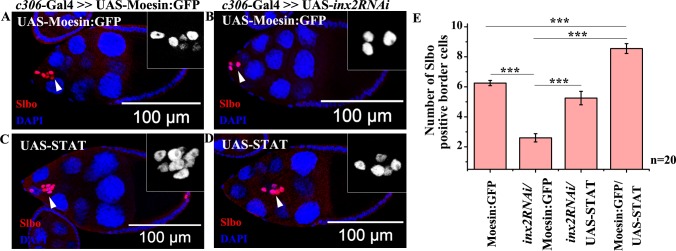
Inx2 dependent loss of BC can be rescued by the overexpression of STAT. (A-D): Stage 9–10 egg chamber of the indicated genotype stained with anti-Slbo antibody (Red) and DAPI (Blue). Arrowheads mark border cell cluster (BC); Inset represents magnified image of BC stained with Slbo. (E): Histogram showing number of BCs in the indicated genotype. Error bar represents Standard Error of Mean. ‘n’ indicates number of egg chambers analyzed. *** p-value <0.001. Note the rescue in number of BCs when STAT is overexpressed in Inx2-depleted clusters.

Since Inx2 down regulation resulted in diminished levels of STAT in the follicle cells, we decided to assess if STAT overexpression can rescue the BC fate in Inx2-depleted follicle cells. To test this we over expressed *inx2RNAi* and STAT in the anterior follicle cells. The control ovaries display 6.25±0.18 border cells (i.e. Slbo positive cells) in the migrating cluster while Inx2 depletion results in smaller clusters with an average of 2.6±0.28 Slbo positive cells only (Fig 3A–3E). Over expression of STAT in otherwise wild type background results in larger clusters with an average of 8.6±0.32 border cells (Fig 3C and 3E). Importantly, STAT overexpression rescued BC fate in the Inx2-depleted follicle cells to near wild type numbers (5.25±0.45 Slbo positive cells) (Fig 3D–3E). While the near complete rescue, suggested that STAT activation is likely to be a limiting factor, it is possible that there are additional targets of Inx2 during BC fate specification. Nevertheless, significant rescue observed upon co-expression of STAT and *inx2RNAi* argues that Inx2 functions upstream of STAT in the JAK-STAT pathway during BC specification. Next question we sought to address was how Inx2 can modulate STAT levels and/or activity in the follicle cells.

### Inx2 modulates domeless receptor internalization in the follicle cells

The strength of the JAK–STAT signaling can be regulated at various levels through distinct mechanisms including interaction(s) with a variety of regulatory proteins that participate in different cellular processes including secretion and endocytosis. For example, ligand dependent internalization of the Upd receptor, Domeless (Dome) is shown to be required for proper activation of JAK-STAT signaling during *Drosophila* oogenesis [19,20].

**Fig 4:**
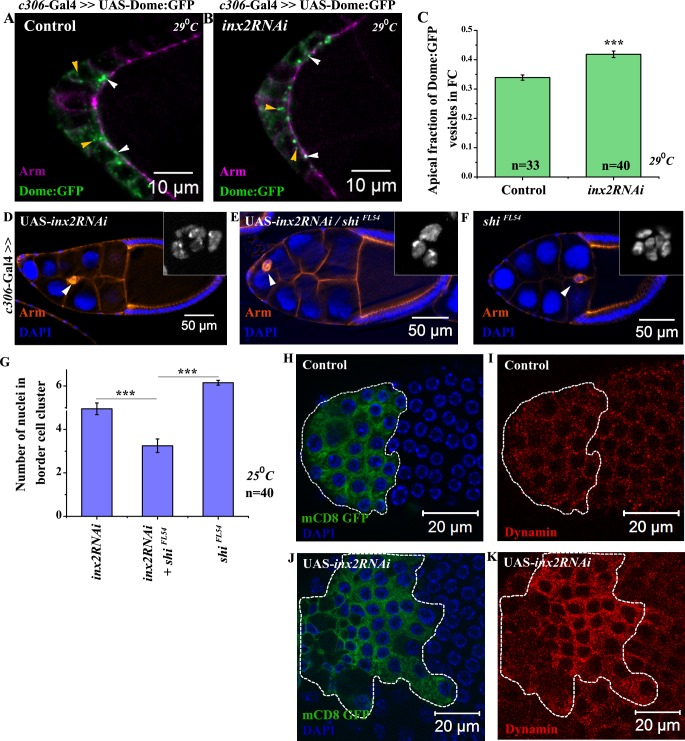
Inx2 regulates Domeless internalization by modulating distribution of Shibire. (A-B): Maximum intensity projected images depict localization of the Dome:GFP vesicles in the follicle cells in control (A) and *inx2RNAi* (B). Anti-Armadillo staining (Magenta) marks the outline of respective egg chamber. White arrowheads indicate vesicles localized to apical membrane. Yellow arrowheads mark cytoplasmic vesicles. (C): Histogram comparing the apical fraction of Dome:GFP vesicles. (D-F): Stage 9–10 egg chambers of indicated genotypes stained with anti-Armadillo (Red) and DAPI (Blue). Inset depicts the number of border cell nuclei. (G): Quantification of border cell nuclei in the migrating cluster for genotype represented in (D-F). Note the enhancement of Inx2 depletion phenotype in Shibire heterozygous background. (H-K): Mosaic analysis employing Actin<Flipout >Gal4 resulting in overexpression GFP alone (H-I) and GFP cum *inx2RNAi* (J-K). Follicle cells overexpressing clones are outlined in white. GFP (Green) (H, J) and anti-Dynamin (Red) (I, K). Note the change in the level and distribution of Shibire (Dynamin) protein in clones over expressing *inx2RNAi* construct compared to nearby wild-type cells and the control clone in (I). Error bar represents Standard Error of Mean. ‘n’ indicates number of egg chambers analyzed. *** p-value <0.001.

This prompted us to examine the spatial distribution of GFP tagged Dome protein in the Inx2-depleted follicle cells. We decided to test if Inx2 modulates the internalization of the Dome receptor in the follicle cells. If this is indeed the case, then perturbing Inx2 activity should also affect the distribution of Dome:GFP in the follicle cells, which ultimately would influence the STAT gradient. To this end, we quantified total number of the Dome:GFP vesicles in the fixed samples from both the wild type and the Inx2-depleted follicle cells. To distinguish the Dome:GFP expression from the background signal reliably, only the vesicles larger than 200μm^2^ and exhibiting an intensity ≥ 50 a.u. were considered for analysis. We observed fewer internalized Dome:GFP vesicles in the Inx2-depleted follicle cells {Average 9 vesicles (8.8±0.43), n = 101 follicle cells} as compared to the wild type {Average 15 vesicles (14.5±0.86), n = 76 follicle cells} (Fig 4A and 4B). To account for this reduction, we also compared the relative distribution of the Dome:GFP vesicles in the experimental and control samples. To estimate the fraction of the apical vesicles in the wild type and Inx2-depleted follicle cells, we stained the egg chambers with anti-Armadillo. Armadillo accumulates at the apical end of the lateral surface and appears as a band around the apical side of the follicle cells [21]. In our analysis, the GFP vesicles that overlapped or were physically associated with Armadillo at the interface of the follicle cells and nurse cells were considered apical. By contrast, the rest of the vesicles that were physically distant from the apical surface were regarded as cytoplasmic. As expected in the Inx2-depleted follicle cells, we observed higher percentage of apical vesicles (42%) compared to control (34%) suggesting that internalization of the Dome receptor is probably compromised (Fig 4C). Though the difference between the control and *inx2RNAi* over expressing samples is relatively modest, it should be noted that *dome* is haploinsufficient and thus Dome activity and/or levels are likely regulated in a stringent manner [19].

To independently validate that vesicular traffic is affected by compromising Inx2 function, we performed the FM4-64 dye labeling and uptake assay for quantifying endocytosis in outer follicle cells [22,23]. Incubation of the egg chambers in live imaging media supplemented with FM4-64 dye labeled the cell membranes and over time FM4-64 was internalized in the form of small vesicles. In wild type follicle cells, the FM4-64 labeled vesicles appeared at the rate of 9±0.3 vesicles/minute while in Inx2-depleted follicle cells, this rate was substantially lower at 5.6±0.5 vesicles/minute (S4A–S4C Fig, S1 and S2 Videos). This observation corroborated that Inx2 modulates the rate of appearance of endocytic vesicles including those containing Dome:GFP in the outer follicle cells. Any alteration in the rate of internalization of Dome:GFP in Inx2-depleted background, in principle, can impair STAT activation and thereby also has the ability to influence BC fate specification.

### Inx2 interacts with shibire and modulates its levels/ distribution in the anterior follicle cells

Since Dome internalization has been shown to be regulated by *Drosophila* Dynamin, Shibire (Shi) [24], we were curious if loss of *inx2* function also influenced *shi* function in BC fate specification. To assess this possibility, we overexpressed *inx2RNAi* both in the *wild type* and *shi*^*FL54*^*/+* anterior follicle cells. (It should be noted that unlike other experiments, this particular genetic interaction was conducted at 25°C to be able to discern either positive or negative interaction between *inx2RNAi* and *shi*). Over expression of *inx2RNAi* in wild type anterior follicle cells results in clusters with 4.95±0.27 number of border cells (Fig 4D). BC specification is further compromised if *inx2RNAi* is overexpressed in *shi*^*FL54*^*/+* background (border cell nuclei 3.25±0.3) suggesting that *inx2* genetically cooperates with *shi* in follicle cells during BC fate specification (Fig 4D–4G).

Next we compared the distribution of Shi protein in wild type and Inx2-depleted follicle cells. In wild type, Shi is localized both in the cytoplasm and at the cell membrane (Fig 4I). Remarkably, we observed significant enrichment in the cytoplasmic levels of Shi in mosaic clones over expressing the *inx2RNAi* (n = 13) compared to the control clones (n>13) (Fig 4K). Quantification of the phenotype suggested that 80% of the *inx2RNAi* overexpressing follicle cells exhibited noticeable enrichment of Shi protein. Similar differences in the cytoplasmic level of Shi:GFP were observed when wild type fusion protein, UAS-Shi:GFP was expressed in the follicle cells in the Inx2-depleted background (S4D–S4H Fig). These observations suggest that Inx2 modulates the distribution and level of Shi in the anterior follicle cells.

Another member of endocytic machinery, Clathrin Heavy Chain (Chc) has been shown to influence Dome internalization [20]. Thus to explore involvement of endocytosis in Inx2 function further we decided to alter Clathrin Heavy Chain (Chc) activity. Supporting the conclusion that Inx2 modulates Dome internalization by influencing endocytic machinery in the follicle cells, over expression of *ChcRNAi* in follicle cells resulted in smaller BC clusters with 3.93±0.24 border cells (S4I–S4K Fig).

Next we sought to determine the possible underlying mechanism(s) that Inx2 might employ to regulate border cell fate in the follicle cells.

### Inx2 mediates the transduction of calcium wave in outer follicle cells

Our data suggests that Shibire localization is sensitive to Inx2 activity. Intriguingly, calcium levels are thought to regulate Shibire distribution and facilitate gap junction mediated intercellular communication [25]. We thus wondered whether Inx2 exerts its influence on Shibire via calcium transport.

**Fig 5:**
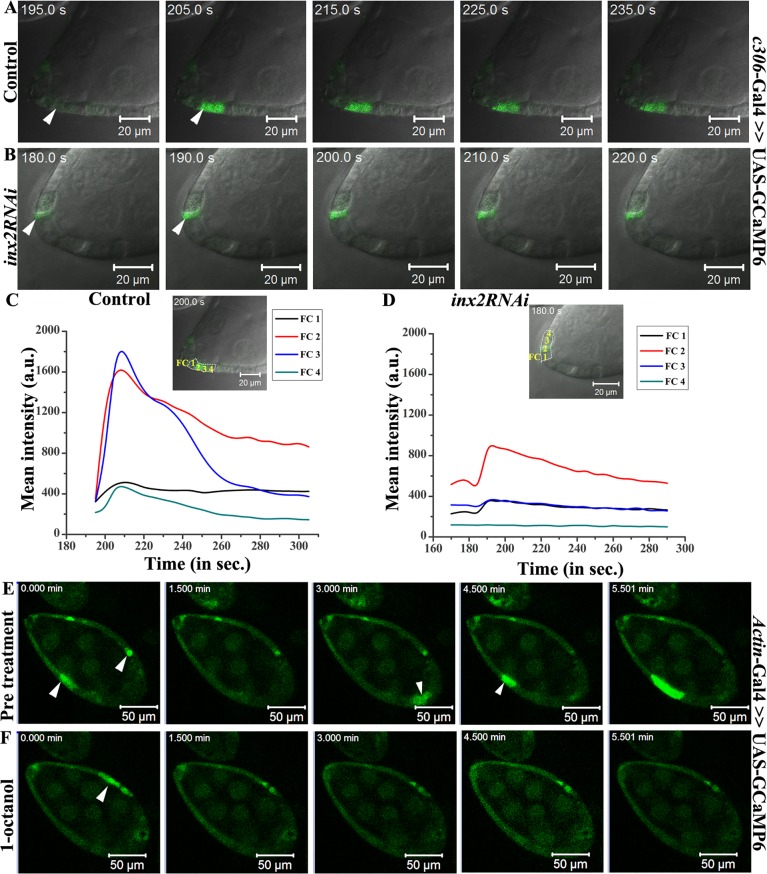
Inx2 modulates Calcium flux in outer follicle cells. (A, B): Snapshots of time lapse imaging of calcium flux in the egg chambers of indicated genotypes. Arrowheads mark the calcium flux. (C, D): GFP intensity calculation for Ca^2+^ levels. Insets show anterior end of egg chamber of control and *inx2RNAi* respectively. 4 consecutive follicle cells (FC1-4) are marked which were considered for calcium flux intensity calculation. Graph represents mean GFP intensity in FC1-4 measured over time (seconds). Note the decrease in the GFP intensity in Inx2-depleted follicle cells (D). (E, F): Snapshots of Ca^2+^ flux in the egg chambers under various conditions. Time interval is denoted in minutes. (E): prior to 1-octanol treatment (F): post 1-octanol treatment. White arrowheads mark Ca^2+^ flux in the follicle cells. Note that incubation in 1-octanol results in the loss of flux in the follicle cells.

As an initial test of this idea we decided to examine if follicle cells display any free calcium. We employed ultra sensitive green fluorescent calcium sensor, GCaMP6, to monitor calcium levels in the outer follicle cells by live cell time-lapse imaging [26]. Curiously we observed periodic wave of free calcium in a random subset of outer follicle cells (Fig 5A and S3 Video). Interestingly, we never observed any initiation or transmission of the calcium flux in the polar cells. Upon closer examination, we noticed that the free calcium wave initiated in a random subset of 1–2 cells, which then spreads to adjacent follicle cells over time (Fig 5A). Since the calcium wave travelled from a given cell to its neighbor, it seemed reasonable that the gap junction proteins expressed in these cells could help establish the connection between the follicle cells to facilitate this movement. Down regulation of Inx2 function in the follicle cells using *c306*-Gal4 driver indeed inhibited the calcium flux (Fig 5B and S4 Video). As can be seen from the calcium reporter intensity plot over a function of time, we observed a drop in its peak intensity in the Inx2-depleted follicle cells compared to control (Fig 5C and 5D). In addition, unlike the control, where the calcium signal moved to the adjacent follicle cell, in Inx2-depleted follicle cells we failed to detect any transmission of calcium signal to the neighbors (Fig 5B and 5D). Moreover, when the egg chambers were incubated with a well-known gap junction-uncoupling agent, 1-octanol, the calcium wave was inhibited supporting the conclusion that the channel activity is required for the calcium flux in the follicle cells (Fig 5E and 5F, S5 and S6 Videos). Similar results were obtained when we incubated the egg chamber with another well-known channel blocker, carbenoxolone [15] (S7 and S8 Videos). Altogether these observations suggested that the gap junction protein Inx2 plays a role both in the activation and mediation of the calcium flux in the somatic follicle cells during *Drosophila* egg chamber development.

Next we decided to explore the source of calcium in the follicle cells that participates in the generation of the flux. Specifically, we sought to determine whether the free Ca^2+^observed in the follicle cells is of extracellular origin or if it was deployed from the intracellular reserves of endoplasmic reticulum. To distinguish between the two possibilities, we analyzed the calcium flux in the presence of drug U-73122 [27]. U-73122 inhibits Phospholipase C (PLC), which is critical for the production of inositol 1,4,5-trisphosphate (IP3). As IP3 stimulates the release of Ca^2+^ from the intracellular stores, we reasoned that application of drug might inhibit the flux in the follicle cells, if the source of free Ca^2+^ was internal. Indeed, upon treatment with 5μM of U-73122, follicle cells that initially exhibited the flux were completely devoid of the signal (S5A–S5D Fig and S9 and S10 Videos). By contrast, application of Dimethyl sulphoxide (DMSO) alone didn’t hinder the flux suggesting that the free Ca^2+^observed in the follicle cells is from the internal stores of the cell and likely not from the extracellular milieu (S5A–S5D Fig).

Since Inx2 depletion affects both the BC fate specification and the calcium transients in the follicle cells, we wondered if these calcium fluxes contribute to the specification of BC fate. To test this we decided to perform a rescue experiment that allowed for increasing the intracellular levels of calcium. We then assessed if such an increase can ameliorate the BC phenotype observed in Inx2-depleted follicle cells.

**Fig 6:**
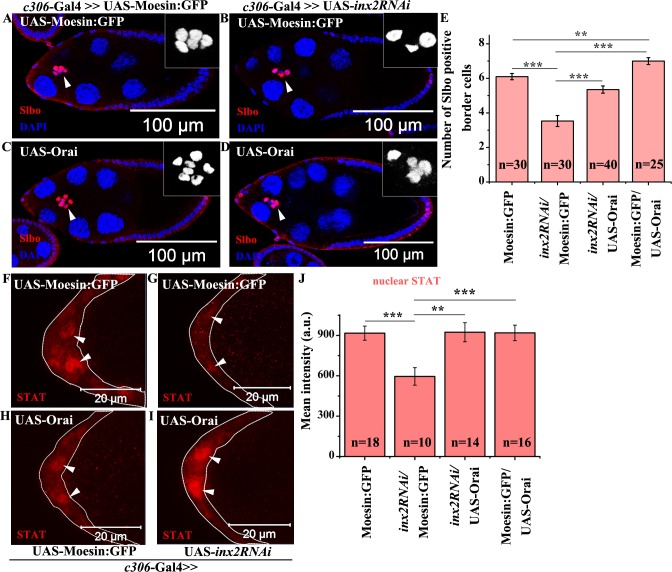
Elevation of Ca^2+^ rescues border cell fate in Inx2-depleted clusters. (A-D): Stage 9–10 egg chamber of indicated genotype stained with anti-Slbo antibody (Red) and DAPI (Blue). Arrowheads mark BC cluster. Inset represents magnified image of BC. (E): Histogram comparing the number of Slbo positive cells in genotypes (A-D). Note the rescue of BC fate in Inx2-depleted cluster over expressing Orai. (F-I): Anterior end of stage 8 egg chamber stained with anti-STAT antibody (Red). Dotted line outlines anterior follicle cells. Arrowheads mark follicle cell nuclei. (J): Quantification of levels of nuclear STAT for genotypes in (F-I). Error bar represents Standard Error of Mean. ** represents p-value <0.01, *** represents p-value <0.001. ‘n’ indicates number of egg chambers evaluated.

Elevation in the level of intracellular free calcium can be achieved either by opening the store operated calcium channel (SOCE) or by increasing the inflow of calcium from the external milieu. Orai, a component of SOCE complex, is a membrane associated calcium channel, that opens under Ca^2+^ depletion thus activating Ca^2+^ entry [28–34]. To test our hypothesis, we co-expressed the *inx2RNAi* along with UAS-Orai in the anterior follicle cells. As observed previously, overexpression of *inx2RNAi* in the anterior follicle cells results in smaller clusters with an average of 3.5±0.32 BCs (Fig 6B and 6E). Interestingly, co-expression of UAS-Orai and *inx2RNAi* resulted in larger cluster with 5.4±0.2 Slbo positive cells, suggesting that free Ca^2+^ functions downstream of Inx2 to regulate BC fate in anterior follicle cells (Fig 6A–6E). In addition over expression of Inositol 1,4,5-tris-phosphate receptor (Itpr) that mediates the release of calcium from intracellular stores also rescues the BC fate in Inx2-depleted follicle cells supporting our conclusion that elevation of calcium levels alone is sufficient to rescue the Inx2 depleted phenotype [35] (S5E Fig related to Fig 6A–6E). We subsequently also addressed if the calcium restores BC fate by elevating STAT levels in Inx2-depleted follicle cells. Consistent with our expectation, we observed higher levels of STAT in the follicle cells overexpressing the *inx2RNAi* and Orai compared to *inx2RNAi* alone suggesting that calcium likely functions via the JAK-STAT pathway to modulate BC fate specification (Fig 6F–6J). This supports the conclusion that rescue achieved by excess Ca^2+^ does not deploy any bypass mechanism and is dependent on JAK-STAT pathway.

**Fig 7:**
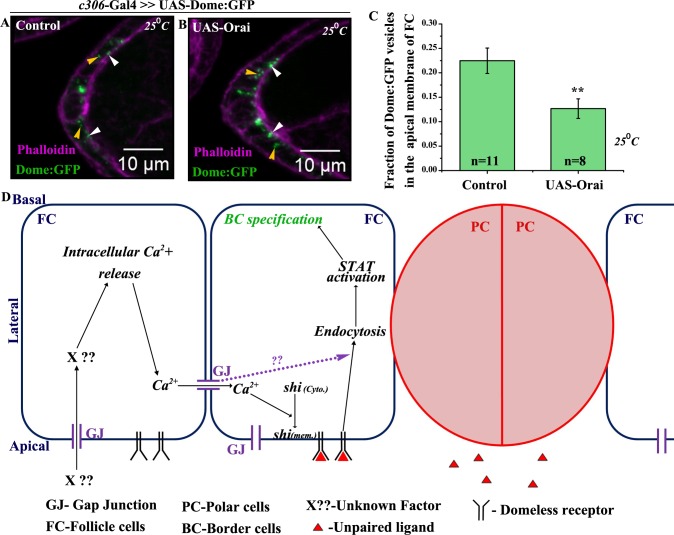
Elevation of Ca^2+^ stimulates vesicle internalization in the follicle cells. (A-B): Maximum intensity projected images depicting the localization of Dome:GFP (Green) vesicles in the anterior follicle cells of indicated genotypes. Rhodamine Phalloidin staining (Magenta) marks the outlines of respective egg chambers. White arrowheads indicate vesicles localized to apical membrane of follicle cells. Yellow arrowheads mark cytoplasmic vesicles. (C): Histogram comparing apical fraction of Dome:GFP vesicles in the follicle cells for genotypes (A, B). (D): Proposed model of Inx2 mediated border cell fate specification. ‘n’ indicates number of egg chambers analyzed. Error bar represents Standard Error of Mean. ** represents p-value <0.01.

Thus, our data argue that calcium functions downstream of Inx2 to regulate BC fate as the phenotype associated with Inx2-depletion can be substantially rescued by increasing the intracellular levels of free calcium. Hence we were curious if the elevation of Ca^2+^ in the follicle cells also leads to stimulation of endocytosis that eventually results in activation of STAT and ultimately leads to BC fate specification. To test this, we analyzed the internalization rate of Dome:GFP vesicles in the follicle cells over expressing Orai. In the fixed samples, we compared the distribution and localization of Dome:GFP vesicles in the control and Orai over expressing follicle cells. We observed lower fraction of apical vesicles of Dome:GFP in the Orai over expressing follicle cells (12.5%) compared to control (22.5%) supporting the conclusion that Orai stimulates Dome:GFP internalization in the follicle cells (Fig 7A–7C). Similar results were also obtained with Clathrin light chain:GFP (Clc:GFP) construct in the follicle cell over expressing Orai (S6A–S6C Fig). We subsequently analyzed endocytosis by live cell imaging in FM4-64 labeled follicle cells over expressing GCaMP6 reporter. Interestingly, we observed several instances where the calcium flux was followed by internalization of labeled vesicles suggesting that the free Ca^2+^ likely stimulates endocytosis in the follicle cells (S6D Fig and S11 Video). Taken together these observations suggest that Inx2 mediates the calcium flux, which in turn probably stimulates endocytosis in the follicle cells.

Based on these data we reasoned that enhancing the levels of endocytosis regulators could potentially modify the BC phenotype induced by Inx2 depletion. Consistently, over expression of Rab5 and Rab7, partially rescued the number of cells recruited to BC fate when Inx2 levels are compromised (S7A–S7E Fig). The modest yet consistent rescue with Rab5 and Rab7 overexpression indicated that Inx2 likely contributes to modulation of the activities of individual endocytosis regulators during border cell fate specification. It is also noteworthy that unlike Rab5 and Rab7, increasing the levels of Rab11 in Inx2-depleted follicle cells didn’t rescue the BC fate appreciably. This observation is consistent with a previous report that JAK-STAT signaling in the follicle cells is independent of recycling endosomes [20].

Since JAK-STAT signaling has been shown to regulate Ca^2+^ levels in Hippocampal neurons, we were curious if similar kind of regulation also existed in the follicle cells [36]. To test this, we co expressed the GCaMP6 reporter and *Janus kinase RNAi (JAKRNAi)* in the follicle cells and examined the calcium flux. Over expression of *JAKRNAi* in the anterior follicle cells impeded border cell movement, however, relatively normal (i.e. similar to the control), calcium flux persisted in the follicle cells. Thus, unlike the hippocampal cells, JAK-STAT signaling does not seem to contribute to the free calcium wave in the follicle cells. (S7F and S7G Fig, S12 Video).

Taken together our results suggest that gap junction protein Inx2 regulates both the transmission and intensity of calcium flux in the outer follicle cells. Since calcium is known to mediate the formation and recruitment of the Dynamin-Calcineurin complex to the endocytic complex, we propose that as the flux moves, it stimulates membrane localization of Shi followed by Dome internalization in the follicle cells [25]. Dome endocytosis subsequently activates STAT in the anterior follicle cells, thus resulting in the specification of BCs (Fig 7D). These data are largely consistent with the model that Inx2 influences calcium flux, which in turn regulates endocytosis. However, at present, we can’t rule out the possibility that Inx2 influences the two events i.e. generation of calcium flux and Domeless receptor endocytosis independently.

## Discussion

Recent observations have suggested that gap junction proteins are involved in regulation of cell fate decisions in various cellular contexts including neuroectoderm differentiation in stem cells, generation of asymmetry in worm neurons and subdivision of neocortex in mammalian brain [1,2,37,38,39]. Our observations have elucidated a possible molecular mechanism underlying function of a gap junction protein involved in cell fate determination. We show that the gap junction protein, Inx2, regulates JAK-STAT pathway in the *Drosophila* follicle cells to specify BC fate. Importantly, our data demonstrate that intercellular communication between the follicle cells is mediated by calcium wave. We show that one of the possible outcomes of calcium flux is stimulation of Dome internalization resulting in STAT activation and BC fate specification (Fig 7D). In this regard it is also important to note that *inx2* transcripts are enriched in the anterior follicle cells, which are the BC precursors [8]. Our results thus provide a novel mechanistic insight into how gap junction mediated intercellular communication can regulate JAK-STAT signaling pathway to ultimately influence BC specification ‬‬‬

### Intercellular communication important for modulation of JAK-STAT pathway

Our study provides novel insight into the nature of intracellular signaling that is activated in gap junction coupled cells during diversification of cell fate. During *Drosophila* oogenesis, BCs are specified in response to Unpaired (Upd) secreted from the polar cells that stimulate JAK-STAT pathway in the neighboring follicle cells. The responding group of follicle cells activate JAK-STAT pathway, and as a result, transform into a migratory population to acquire the BC fate. Our results suggest that one of the outcomes of intercellular communication mediated by transmission of calcium wave is ultimately crucial for the STAT activation and specification of BCs. Similar horizontal inputs from other cascades could prove to be a general theme in coordinating growth and development during pattern formation in a variety of multicellular organisms.

Several reports have indicated that intercellular communication could be critical for *Drosophila* oogenesis [40]. For the first time, our results with GCaMP6 reporter demonstrate that the follicle cells, in fact, communicate via a calcium wave. Though at present we don’t understand the mechanism underlying the initiation of the wave i.e. calcium flux, an attractive possibility in this regard is potential signaling from the nurse cells. Two observations argue in favor of this option. First, down regulation of Inx2 function in the follicle cells hinders the flux intensity. Second, it has been reported that Inx2 in the follicle cells is localized at very close proximity to Inx4 in the nurse cells [13]. Since the apical surface of the follicle cells is in close contact with the nurse cells, signal(s) from the germ line can possibly activate the calcium flux. As germ line clones for Inx2 didn’t influence BC fate, it is likely that Inx2 plays a significant role only in the follicle cells where as another germ line specific gap junction family protein may partner with Inx2. Interestingly, gap junction mediated germline-soma interaction of this nature has been reported in *Drosophila* spermatogenesis [41]. In this instance, Inx2 from the somatic cells and Inx4 from male germ line form a heterotypic channel critical for mediating effective germline-soma communication during spermatogenesis. Our results suggest the possibility of similar heterotypic interaction between Inx2 and Inx4 during oogenesis and future experiments will explore this avenue in detail.

### Positive influence of endocytosis on JAK-STAT pathway in the follicle cells

Internalization of the JAK-STAT receptor ligand complex and their trafficking through the endosomal compartment is critical for STAT activation [20]. By contrast, another study in *Drosophila* imaginal discs suggests that down regulation of the endocytic components upregulates the JAK-STAT pathway [42]. Our findings establish that vesicle internalization in the follicle cells is important for potentiating JAK-STAT activity. Importantly, our data suggest an unanticipated functional loop involving gap junction protein Inx2, endocytic machinery component Shi and, calcium flux during border cell specification. It will be of interest to determine whether Inx2 mediated calcium flux is directly responsible in changing endocytosis and future experiments will test whether the effect of Inx2 on calcium levels can be functionally uncoupled from its impact on endocytosis.

In distinct developmental contexts, several signaling pathways are reiteratively deployed to achieve remarkably diverse outcomes. For instance, signaling ligands such as Wnt and Hedgehog not only control cell fate specification but can also moonlight as guidance cues. The signaling machinery components adopt both canonical and non-canonical modes of signal transduction in a context dependent manner. Thus in order to be able to modulate the signaling pathways, several mechanisms ought to be in place that either potentiate or dampen the response to achieve the appropriate outcome. To name a few, post-translational modifications, nuclear transport, protein degradation and controlled release via secretion are among numerous strategies, which are employed for this purpose. In a number of different contexts, the components of the endocytosis machinery as well as calcium have been reported to be major players involved in the modulation of the signaling pathways. Future studies should focus on how the endocytic regulators affect activity of JAK-STAT signaling in different systems to elicit qualitatively distinct responses.

### Gap junction proteins: unique conduits during development and disease

For any multicellular organism, proper tissue differentiation and organogenesis are two critical aspects during early embryonic and subsequent adult development. Though gap junctions serve at critical junctures during organismal development, recent studies have revealed an unexpected functional link between gap junction proteins and progression of various cancers [43–46]. Since tumor progression has been closely linked with aberrant differentiation, it would be interesting to examine if loss of cellular identity in tumorigenesis is causally related to impaired gap junction function. That such a connection is indeed a possibility was suggested by recent studies on colon cancer cells, where restoring the gap junction function could reestablish the differentiation [47]. It will be interesting to examine if intercellular communication mediated by gap junctions is responsible to acquire and/or maintain their epithelial fate. Future studies will thus focus on mechanistic underpinnings of involvement of gap junction proteins in cell fate specification in both normal and disease contexts.

## Materials and Methods

### *Drosophila* stocks and genetics

Fly stocks and crosses were maintained under standard conditions (25°C) unless otherwise stated. *c306*-Gal4, *Actin*-Gal4 and *Upd*-Gal4 used for the expression of various transgenes were crossed with UAS-GFP reporter to test if the drivers were functional. P{UAS-Inx2}, P{UAS-RFP:Inx2}, P{UAS-Inx2:RFP} were kindly provided by Dr Andrea Brand. P{UAS-Inx2:GFP} and P{UAS-Inx2} were also provided by Reinhard Bauer (University of Bonn, Germany). P{UAS-Domeless:GFP} was obtained from Prof. Stéphane Noselli (Institute of Biology Valrose, France). P{UAS-Itpr} and P{UAS-Orai} were received from Prof. Gaiti Hasan (NCBS, India). *inx2* alleles P{w[+mC] = lacW}*inx2*^***G0173a***^ (DGRC-111858), P{w[+mC] = lacW}*inx2*^***G0059***^ (DGRC-114609) were obtained from Drosophila Genetic Resource Centre, Kyoto. Rest of the fly lines including UAS-*inx2RNAi* {P(TRiP.JF02446), Bl- 29306} and P {EPgy2}Stat92E^EY14209^/TM3Sb Ser (Bl-20915) line were obtained from Bloomington Drosophila Stock Centre, Indiana University. It should be noted that this particular *inx2RNAi* construct hasn’t been predicted to have off targets based on in-silico methods [15].

### Immunohistochemistry

Ovary dissection, fixation and staining were performed using standard protocol [48]. The following primary antibodies were used: mouse anti-Armadillo [Developmental Studies Hybridoma Bank (DSHB), 1:25, N27A1]; rat anti-Slbo 1:400 (gift from P. Rorth); rabbit anti-STAT 1:700 (gift from Steven Hou), rabbit anti-GFP 1:1000 (Life technologies), guinea pig anti-Inx2 1:1000 [41] and mouse anti-Dynamin 1:500 (BD, gift from Dr Richa Rikhy). Secondary antibodies conjugated with Alexa-488 and Alexa-568 (Molecular Probes) were used at 1:250 and 1:400 dilutions respectively. Rhodamine Phalloidin staining was performed using standard procedure [48].

### Statistics

Two-tailed Test of unequal variance was used in Excel to assess statistical significance. Graphs were plotted using Origin Pro 8. * Indicates p value <0.05, ** indicates <0.01, *** indicates <0.001. We also carried out non-parametric Mann-Whitney test (employing Past3 application), which is used for populations that are not normally distributed. Consistent with the T Test results, we observed statistically significant differences with Mann-Whitney test too. Standard Error of Mean (S.E.M.) was calculated and represented for each data.

### Fly genetics and mutant analysis

For mutant analysis, P{w[+mC] = lacW}*inx2*^***G0173a***^ FRT 19A/FM7 flies were crossed with hsFLP,tubP-Gal80,neo FRT19A; *Actin-*Gal4 UASp-Moesin:Cherry/TM3Sb. F1 flies were subjected to heat-shock for 1 hour thrice a day for 3 consecutive days. Flies were incubated at 25°C for 5 days followed by fattening at 25°C. For MARCM rescue experiment UAS-Inx2 was combined with P{w[+mC] = lacW}*inx2*^***G0173a***^ FRT 19A/FM7 stock and crossed as mentioned earlier.

For flip out clonal analysis, flies of genotype hsFLP UAS-mCD8: GFP; Actin <flipout>Gal4/ UAS-*inx2RNAi* were subjected to heat-shock for 1 hour duration thrice a day for 3 consecutive days. The flies were dissected on the 6^th^ day after the last heat shock and ovaries were immunostained with Inx2 or Dynamin antibody.

For experiments in which the F1 genotype was lethal (*ChcRNAi* with *c306-*Gal4), temperature-sensitive Gal80 was used to downregulate Gal4 activity during early development. Crosses were kept at 16°C till progenies emerged. F1 flies were shifted to 31°C (restrictive temperature) for 18 hours to inactivate Gal80 before dissection.

In all of the RNAi experiments, desired F1 genotype flies were fattened at 29°C for 18 hrs. UAS-Moesin:GFP [49] or UAS-mCD8:GFP were used to normalize the number of UAS constructs in the cross. All imaging were performed with Apotome & LSM 710 confocal microscope.

### Quantification of BC in the cluster

To quantify the number of nuclei, only stage 9–10 egg chambers, where BC cluster had detached, were considered. For scoring migration defect, stage 10 egg chambers were identified and quantification carried out as reported previously [48]. Based on the distribution of migration efficiency, adequate weightage was given to each phenotypic class for quantifying the BC nuclei.

### Calculation of nuclear STAT intensity

From the Z-stacks of the anterior end of stage 8 egg chambers, a region of interest adjacent to the polar cells was selected and the outlines of the cells were marked. The three dimensional extent of each nucleus, corresponding to its height in z direction was noted down, and those particular z-planes bearing the cells were extracted separately. From these planes the cumulative mean intensity of STAT was calculated using software (Zen 2010) by examining a maximum intensity projection (MIP) image for each cell.

### Quantification of Slbo positive i.e. border cells

Numbers of Slbo positive cells were quantified from stage 9–10 egg chambers. All reference images were captured in single plane and high magnification inset images in the figures are maximum intensity projection of whole cluster in 2D.

### 10XSTAT GFP intensity calculation

For the quantification of *10XSTAT-GFP* in the follicle cells anterior end of stage 8 egg chambers were captured keeping polar cells at the center plane. For intensity calculation, cells were outlined on the basis of Armadillo staining and the mean intensity of STAT GFP was extracted. For quantifying *10XSTAT-GFP* in migrating border cell cluster, various Z stacks were captured for the whole border cell cluster. The 3D images were projected in 2D employing maximum intensity projection (MIP) algorithm of Zen 2010. In the MIP images the border cell clusters were outlined with reference to Armadillo staining and the mean intensity of STAT GFP was extracted.

### Domeless GFP vesicle count

Egg chambers were stained with Armadillo or Phalloidin and anti-GFP antibody. Imaging was performed in Apotome microscope with 40X Plano-Apochromat objective (0.95 N.A.) by keeping polar cells at the center with 340nm z interval. Vesicle quantification was done for each z plane manually or using cell counter plugin from Image J. Vesicles that overlapped in successive z planes were considered only once. Armadillo or Rhodamine Phalloidin was used to label the follicle cell membrane.

### Clc: GFP vesicle count

Egg chambers were stained with Phalloidin and anti-GFP antibody. Imaging was performed in Apotome microscope with 40X Plano-Apochromat objective, (0.95 N.A.) by keeping polar cells at the center with 340nm z interval. Vesicle quantification was done for each z plane manually. Vesicles that overlapped in successive z planes were considered only once. Rhodamine Phalloidin was used to label the follicle cell membrane.

### Endocytosis imaging

Flies were dissected in live imaging media {Schneider medium supplemented with 15% FBS, 0.5mg/ml insulin (sigma)} and incubated in media supplemented with FM4-64 dye (life technologies) (10μg/ml in S2 media) for 2 minutes [50]. Egg chambers were transferred to fresh Schneider media and immediately mounted on a glass bottom dish coated with poly-D-lysine (sigma) [50]. The anterior part of the stage 8-egg chamber was captured with polar cells marking the central z plane. 5Z stacks were captured, equally distributed on both sides of the central z plane under the confocal microscope CLSM 710 (Carl Zeiss, Germany) with a Plan-Apochromat 100X oil immersion objective (N.A. 1.4). The z intervals were 740nm apart and 12 bit images of frame size 512X512 were captured. This was followed by time lapse imaging with 3.15μs pixel dwell having time interval of 15.0 seconds per frame. In Zen 2010, a median filter (3X3) was applied to lower the noise in the image sequences before vesicle counting was performed manually for each z stack. Newly formed vesicles were counted in each frame and 3 cells on either side of the polar cells were included. Each vesicle was considered only once.

### Calcium imaging and FM4-64 internalization assay

P{UAS-GCaMP6m} (Bl-42748) line was over expressed by *c306*-Gal4 or *Actin*-Gal4 for detecting free Ca^2+^ in the follicle cells. 12 bit images of frame size 512X512 were captured with CLSM 710 (Zeiss) with Plan-Apochromat 63X oil immersion objectives (N.A. 1.4). Argon laser line 488 nm was used for GCaMP6 reporter and DPSS 561 nm laser employed for FM4-64 dye (pinhole diameter 187 μm). Fast continuous live imaging with 6.3 μm pixel dwell at frame interval 5 seconds for 5–6 minutes was used for recording the calcium flux alone. Sequential imaging was performed using Argon 488 laser and DPSS 561 nm laser with time interval 7 seconds per frame for recording calcium flux and FM4-64 dye internalization.

For calcium flux graph, one Region of Interest (ROI) was chosen and the mean intensity of the signal in each follicle cell through which the flux moves was plotted against time.

### PLC inhibitor treatment

After egg chambers were imaged for 5 minutes to record the calcium flux they were treated for 15 minutes with live imaging media containing 5μM PLC inhibitor (or DMSO control). Post treatment with PLC inhibitor, the calcium flux was again recorded for the same Region of Interest.

### 1-octanol treatment

The pretreatment flux was recorded for the egg chambers prior to incubation with 2mM 1-octanol (Sigma-297887). After 15 minutes of incubation in 1-octanol, the egg chamber was washed and imaged in normal live imaging media.

### Carbenoxolone treatment

Control calcium flux was recorded for the egg chamber just after the addition of 125μM carbenoxolone (Sigma-C4790) to the live imaging media. After 20 minutes of incubation, calcium flux was again recorded for the same egg chamber.

### Quantification of shibire: GFP vesicles

The entire anterior end of stage 10 egg was captured using the Z stack imaging. The 3D images were projected in 2D employing maximum intensity projection (MIP) algorithm and cells expressing Shibire:GFP were outlined. The GFP vesicles greater than 0.25μm diameters in size were quantified using cell counter plugin in Image J. Number of vesicles per unit area (per 100 μm^2^) was used to compare the control and *inx2RNAi* expressing follicle cells.

## Supporting Information

S1 Fig Channel activity of Inx2 modulates border cell fate in the follicle cells. (A): Histogram compares the area of the control and Inx2-depleted border cell cluster (in μm^2^). (B-E): Single plane image of stage 9–10 egg chamber of indicated genotype stained with anti-Armadillo antibody (Red) and DAPI (Blue). Inset represents magnified image of BC nuclei. Arrowheads mark border cell cluster. (F): Quantification of border cells for the genotypes (B-E). Note the rescue in the number of cells in migrating cluster when Inx2cDNA and Inx2:RFP are overexpressed in Inx2-depleted follicle cells. (G, H, J, K): Single plane image of egg chamber of indicated genotype stained with DAPI (Blue), anti-Slbo (Green) (G, H) and anti-Armadillo (Red) (J, K). Arrowheads mark the border cell cluster. Inset represents the DAPI staining in (G, H). (I): Histogram compares the number of border cells in the control (G) and RFP:Inx2 (H) overexpressing clusters. (L): Histogram compares the migration efficiency for control (J) and *inx2RNAi* (K) border cell cluster respectively. ‘n’ indicates number of egg chambers analyzed. Error bar represents Standard Error of Mean. *** represents p-value <0.001.
(TIF)

S2 Fig Down regulation of Inx2 function in polar cells doesn’t affect border cell fate specification. (A, B): Stage 8 chamber of the indicated genotypes stained with anti-Inx2 (Red) and DAPI (Blue). (A’, B’): Magnified image of the anterior of the egg chamber shown in (A) and (B) respectively. Arrowhead marks the interface of two polar cells in (A’) and (B’). Note the absence of punctate staining for Inx2 in (B’) compared to (A’). (C, D): Single plane image of stage 9–10 egg chamber of indicated genotype stained with anti-Armadillo antibody (Red). Inset represents magnified image of BC nuclei in DAPI. Arrowheads mark border cell cluster. (E): Quantification of number of nuclei in border cell cluster of indicated genotype in (C) and (D). ‘n’ indicates the number of egg chambers analyzed. n.s. stands for statistically not significant.
(TIF)

S3 Fig Inx2 affect the *10XSTAT-GFP* expression in border cell cluster. (A, B): Single plane image of stage 9–10 egg chambers of indicated genotypes stained with anti-Armadillo antibody (Red) and GFP (Green). Arrowheads mark border cell cluster. (C-F): Maximum intensity projections of border cell cluster shown in (A) and (B). Control (C, D) and *inx2RNAi* (E, F) stained with anti-Armadillo (Red) and GFP (Green). (G): Histogram displays difference in the intensity level of *10XSTAT-GFP* in control (D) and *inx2RNAi* (F) border cell cluster respectively. ‘n’ indicates number of egg chambers analyzed. Error bar represents Standard Error of Mean. ** represents p-value <0.01.
(TIF)

S4 Fig Inx2 regulates vesicles internalization and Shibire localization. (A, B): Snapshot of time-lapse imaging of follicle cells of stage 8 egg chambers labeled with lipophilic dye FM4-64 (Red). Time interval is indicated in minutes (min). 0 min is the merged image of the Moesin:GFP and FM4-64 for the indicated genotypes. White arrows mark newly formed vesicle at the apical end. (C): Histogram compares the rate of appearance of vesicles per minute for genotypes indicated in (A) and (B). (D-G): Overexpression of UAS-ShiWT:GFP by *c306*-Gal4 driver in wild type (D, E) and Inx2-depleted (F, G) follicle cells. Follicle cell overexpressing ShiWT:GFP are outlined in white. GFP (Green) (D, F) and anti-Armadillo (Red) (E, G). Arrowheads mark the Shibire:GFP puncta. Note higher number of Shibire:GFP puncta in Inx2-depleted follicle cells compared to the control. (H). Quantification of Shibire:GFP puncta for genotypes represented in (D) and (F). (I, J) Stage 9–10 egg chamber of indicated genotype stained with anti-Slbo (Red) and DAPI (Blue). Arrowheads mark BC cluster and inset shows Slbo staining of the border cell cluster. (K) Histogram depicting the comparison of Slbo positive cells for genotypes represented in (I) and (J). ‘n’ indicates number of egg chambers analyzed. Error bar represents Standard Error of Mean. *** represents p-value <0.001.
(TIF)

S5 Fig Intracellular calcium in the follicle cells regulates the border cell fate specification. (A-D): Snapshots of Ca^2+^ flux in the egg chambers of indicated genotype under various conditions. Time is denoted in seconds. (A, B): pre- and post-DMSO treatment. (C, D): pre- and post-PLC inhibitor (PLCi) treatment. White arrowheads mark Ca^2+^ flux. The observed flux in the follicle cells is due to the release of intracellular Ca^2+^. (E): Histogram representing number of Slbo positive border cells in the indicated UAS lines driven by *c306*-Gal4. Note the rescue in border cell fate in Inx2-depleted clusters overexpressing UAS-Itpr. ‘n’ indicates number of egg chambers analyzed. Error bar represents Standard Error of Mean. *** represents p-value <0.001.
(TIF)

S6 Fig Increase in calcium level triggers vesicle internalization. (A, B): Maximum intensity projection of egg chambers of indicated genotypes showing cellular distribution of Clathrin light chain GFP (Clc:GFP) vesicles in the anterior follicle cells. Rhodamine Phalloidin (Magenta) marks the outline of the egg chamber. White arrowheads indicate vesicles localized to apical membrane of follicle cells. Yellow arrowheads mark cytoplasmic vesicles. (C): Histogram compares apical fraction of Clc-GFP vesicles in the control and UAS-Orai over expressing follicle cells. (D): Snapshot of time lapse imaging of calcium flux in the presence of FM4-64 dye. Arrows mark the follicle cells with active calcium flux and it correlates with FM4-64 dye internalization. ‘n’ indicates number of egg chamber analyzed. Error bar represents Standard Error of Mean. *** represents p-value <0.001.
(TIF)

S7 Fig Overexpression of Rabs rescues border cell fate in Inx2–depleted follicle cells. (A-D) Stage 9–10 egg chamber of indicated genotypes stained with anti-Armadillo (Red). Arrowheads mark border cell cluster and inset shows DAPI staining of border cell cluster. (E): Histogram showing number of nuclei in border cell clusters in the indicated genotypes. Note the rescue in BC number when endocytosis regulators are overexpressed. (F, G): Snapshots of time lapse imaging of calcium flux in control (*c306*-Gal4; UAS-GCaMP6) and *Janus Kinase (JAK) RNAi (c306*-Gal4; UAS-GCaMP6/ UAS-*JAKRNAi*) follicle cells. Arrowheads mark the calcium flux. No appreciable difference in the calcium flux was observed in follicle cells overexpressing *JAKRNAi*. ‘n’ indicates number of egg chamber analyzed. Error bar represents Standard Error of Mean. *** represents p-value <0.001, * represent p-value <0.05.
(TIF)

S1 Video Visualizing endocytosis using FM4-64 labeled vesicles. Time lapse imaging of anterior follicle cells in a control egg chamber of the genotype *c306*-Gal4; UAS-Moesin:GFP stained with membrane dye FM4-64 (Red). Green is Moesin:GFP expression that outlines the anterior follicle cells. Note the appearance of new FM4-64 labeled vesicles (Red) in the follicle cells. Time is denoted in minutes. Arrow marks the appearing vesicles.
(MOV)

S2 Video Inx2 depletion impedes the appearance of newly formed vesicles. Time lapse imaging of the anterior follicle cells. Genotype: *c306*-Gal4; UAS-*inx2RNAi*/ UAS-Moesin:GFP. FM4-64 dye (Red) labels the membrane. Green is Moesin:GFP expression that outlines the anterior follicle cells. Time is denoted in minutes. Arrow marks the appearing vesicles.
(MOV)

S3 Video Follicle cells exhibit calcium wave. Time lapse imaging of an egg chamber (Nomarski) expressing genetically encoded calcium indicator (Green). Genotype: *c306*-Gal4; UAS-GCaMP6. Note the random subset of follicle cells exhibiting the calcium flux (Green). Time is denoted in seconds.
(MOV)

S4 Video Inx2 mediates the calcium flux in the follicle cells. Time lapse imaging of an egg chamber (Nomarski) expressing genetically encoded calcium indicator (Green). Genotype: *c306*-Gal4; UAS-GCaMP6/ UAS-*inx2RNAi*. Intensity and transduction of calcium flux (Green) in the follicle cells is appreciably decreased. Time is denoted in seconds.
(MOV)

S5 Video Follicle cells exhibiting calcium flux before treatment with 1-octanol. Time lapse imaging of an egg chamber (Nomarski) prior to the treatment with 1-octanol. Genotype: *Actin*-Gal4; UAS-GCaMP6. The calcium flux is observed in the follicle cells. Time is denoted in minutes.
(MOV)

S6 Video 1-octanol impedes calcium flux in the follicle cells. Time lapse imaging of the same egg chamber captured in S5 Video, after treatment with 1-octanol. Note that intensity and transduction of calcium flux (Green) in the follicle cells is appreciably decreased. Time is denoted in minutes.
(MOV)

S7 Video Control follicle cells exhibiting calcium flux. Time lapse imaging of an egg chamber (Nomarski) just after the addition of carbenoxolone to medium (0 time point). Genotype: *c306*-Gal4; UAS-GCaMP6. The calcium flux is observed in the follicle cells. Time is denoted in seconds.
(MOV)

S8 Video Carbenoxolone impedes calcium flux in the follicle cells. Time lapse imaging of the same egg chamber captured in S7 Video after incubation with carbenoxolone for 20 minutes. Genotype: *c306*-Gal4; UAS-GCaMP6. Note that intensity and transduction of calcium flux (Green) in the follicle cells is decreased. Time is denoted in seconds.
(MOV)

S9 Video Control; Source of Calcium is from the internal stores of the follicle cells. Time lapse imaging of an egg chamber (Nomarski) prior to the treatment with PLC inhibitor. Genotype: *c306*-Gal4; UAS-GCaMP6. The calcium flux is observed in the follicle cells. Time is denoted in seconds.
(MOV)

S10 Video Source of Calcium is from the internal stores of the follicle cells. Time lapse imaging of the same egg chamber captured in S9 Video, after treatment with PLC inhibitor. Note the loss of calcium flux in the follicle cells. Time is denoted in seconds.
(MOV)

S11 Video Calcium flux stimulates dye internalization. Sequential time- lapse imaging of the egg chamber of the genotype *c306*-Gal4; UAS-GCaMP6 (Magenta) stained with membrane dye, FM4-64 dye (Cyan). Internalization of FM4-64 dye is readily visible in the follicle cells (Arrows) that exhibit the calcium flux. Time is denoted in minutes.
(MOV)

S12 Video Calcium flux is independent of JAK-STAT signaling. Time lapse imaging of the experimental egg chamber (Nomarski). Genotype:*c306*-Gal4;UAS-GCaMP6/ UAS-*JAKRNAi*. Calcium flux (Green) is present in the follicle cells overexpressing the *JAKRNAi*. Time is denoted in seconds.
(MOV)
