# Supplementary figures and images for: A Gap Junction Protein, Inx2, Modulates Calcium Flux to Specify Border Cell Fate during Drosophila oogenesis
Source: PLoS Genet. 2017 Jan 23;13(1):e1006542. doi: 10.1371/journal.pgen.1006542 (PMC5256874; doi:10.1371/journal.pgen.1006542)

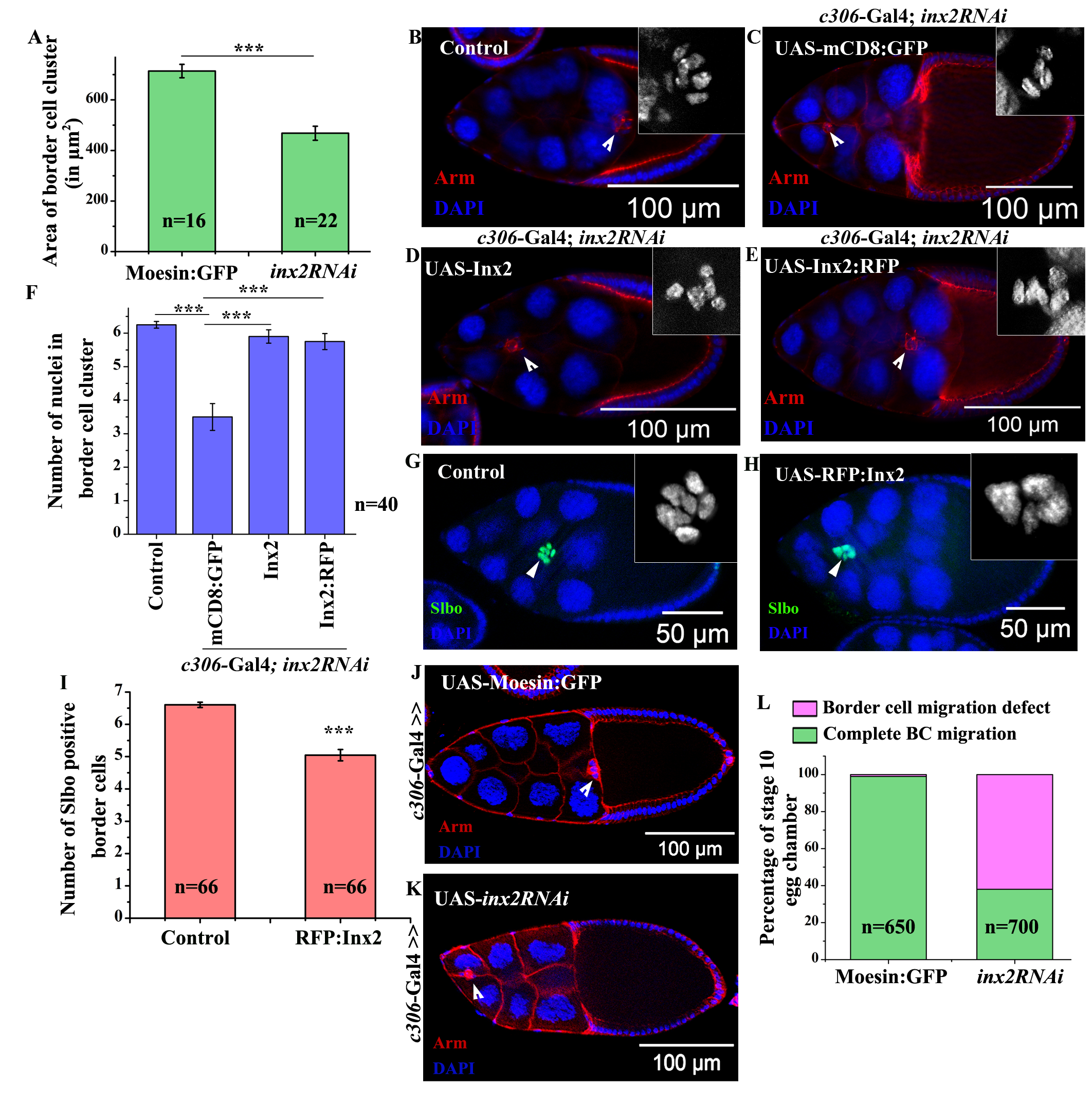

Supplement: S1 Fig — (A): Histogram compares the area of the control and Inx2-depleted border cell cluster (in μm2). (B-E): Single plane image of stage 9–10 egg chamber of indicated genotype stained with anti-Armadillo antibody (Red) and DAPI (Blue). Inset represents magnified image of BC nuclei. Arrowheads mark border cell cluster. (F): Quantification of border cells for the genotypes (B-E). Note the rescue in the number of cells in migrating cluster when Inx2cDNA and Inx2:RFP are overexpressed in Inx2-depleted follicle cells. (G, H, J, K): Single plane image of egg chamber of indicated genotype stained with DAPI (Blue), anti-Slbo (Green) (G, H) and anti-Armadillo (Red) (J, K). Arrowheads mark the border cell cluster. Inset represents the DAPI staining in (G, H). (I): Histogram compares the number of border cells in the control (G) and RFP:Inx2 (H) overexpressing clusters. (L): Histogram compares the migration efficiency for control (J) and inx2RNAi (K) border cell cluster respectively. ‘n’ indicates number of egg chambers analyzed. Error bar represents Standard Error of Mean. *** represents p-value <0.001. (TIF) [file pgen.1006542.s001.tif]

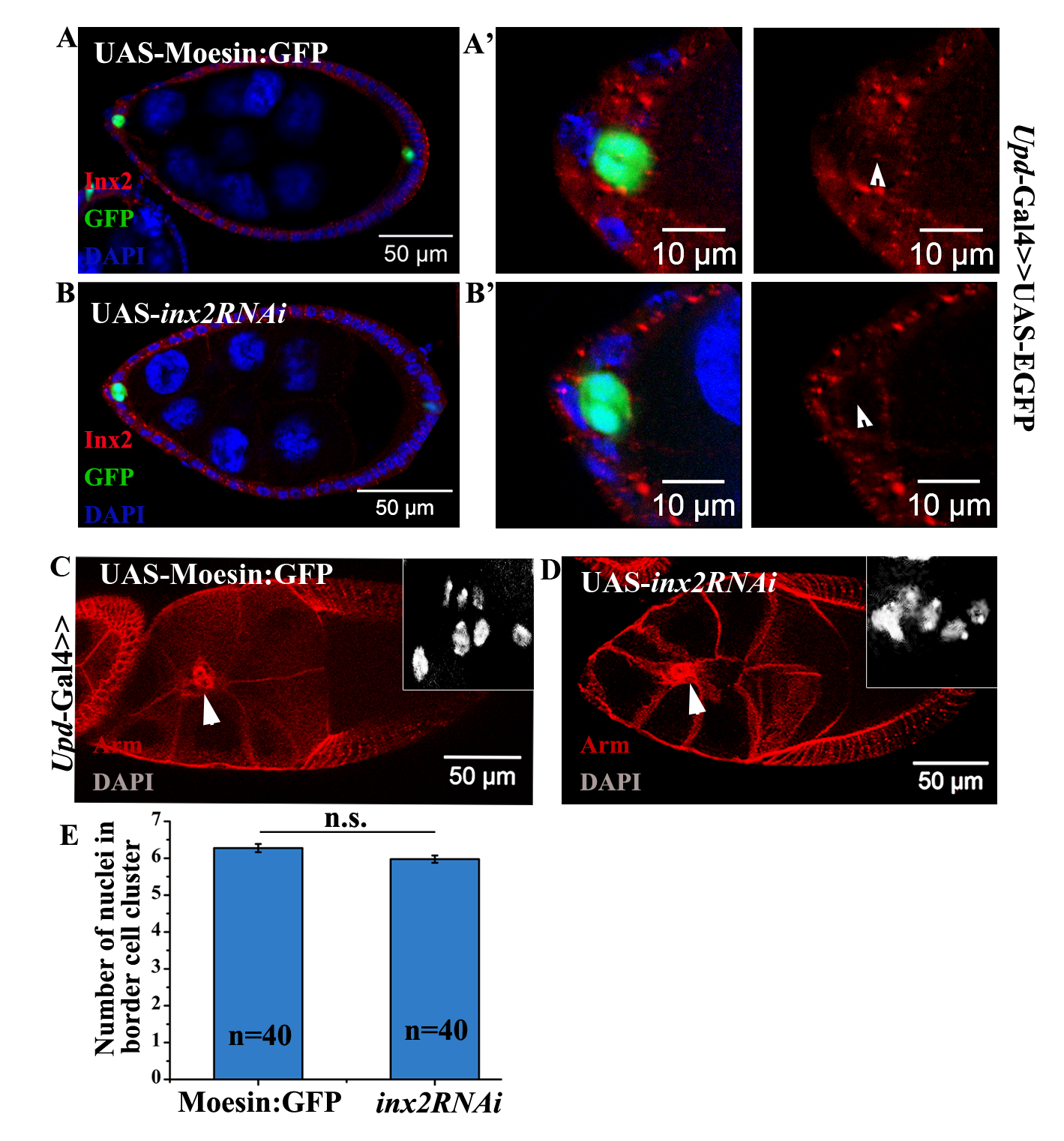

Supplement: S2 Fig — (A, B): Stage 8 chamber of the indicated genotypes stained with anti-Inx2 (Red) and DAPI (Blue). (A’, B’): Magnified image of the anterior of the egg chamber shown in (A) and (B) respectively. Arrowhead marks the interface of two polar cells in (A’) and (B’). Note the absence of punctate staining for Inx2 in (B’) compared to (A’). (C, D): Single plane image of stage 9–10 egg chamber of indicated genotype stained with anti-Armadillo antibody (Red). Inset represents magnified image of BC nuclei in DAPI. Arrowheads mark border cell cluster. (E): Quantification of number of nuclei in border cell cluster of indicated genotype in (C) and (D). ‘n’ indicates the number of egg chambers analyzed. n.s. stands for statistically not significant. (TIF) [file pgen.1006542.s002.tif]

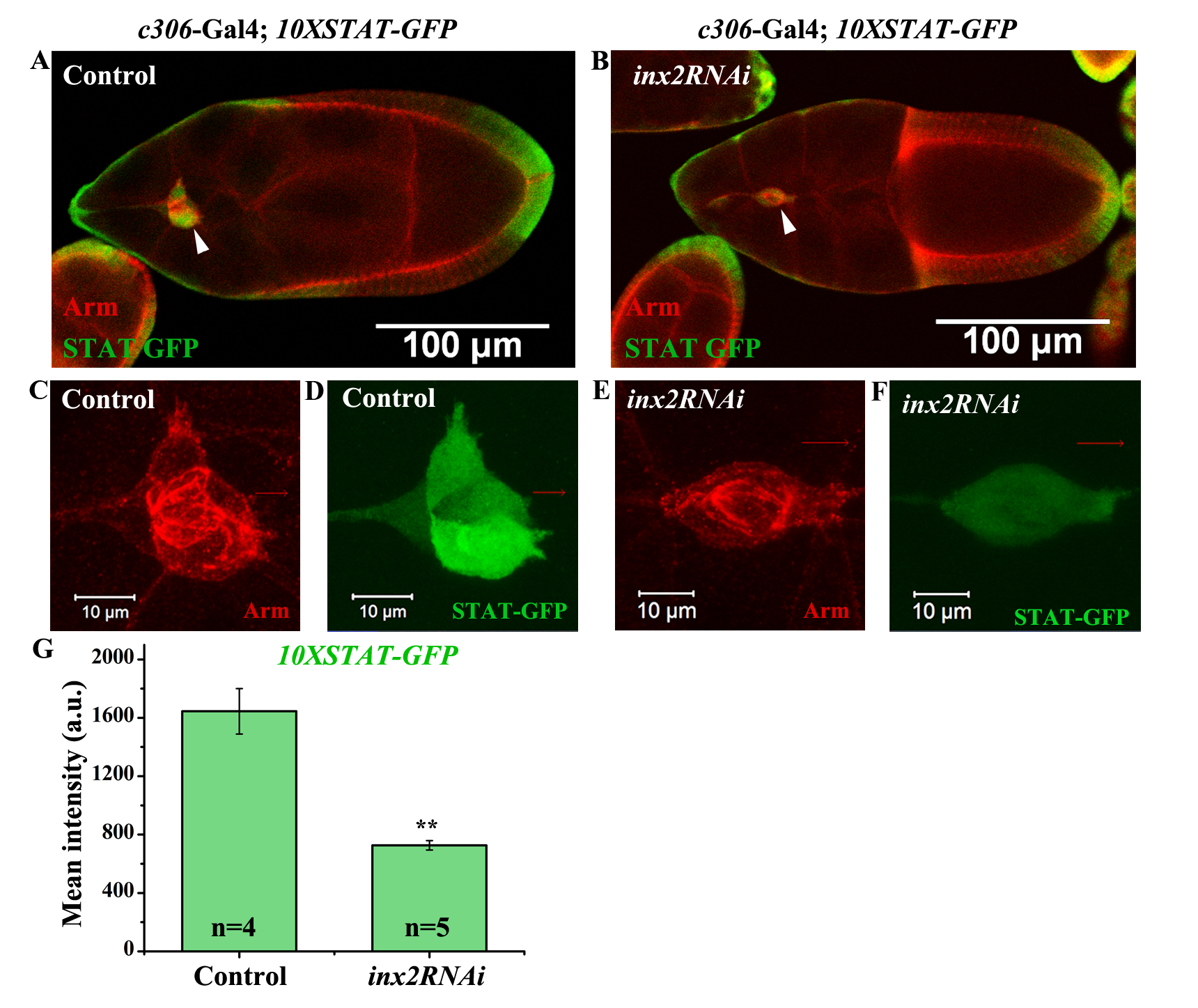

Supplement: S3 Fig — (A, B): Single plane image of stage 9–10 egg chambers of indicated genotypes stained with anti-Armadillo antibody (Red) and GFP (Green). Arrowheads mark border cell cluster. (C-F): Maximum intensity projections of border cell cluster shown in (A) and (B). Control (C, D) and inx2RNAi (E, F) stained with anti-Armadillo (Red) and GFP (Green). (G): Histogram displays difference in the intensity level of 10XSTAT-GFP in control (D) and inx2RNAi (F) border cell cluster respectively. ‘n’ indicates number of egg chambers analyzed. Error bar represents Standard Error of Mean. ** represents p-value <0.01. (TIF) [file pgen.1006542.s003.tif]

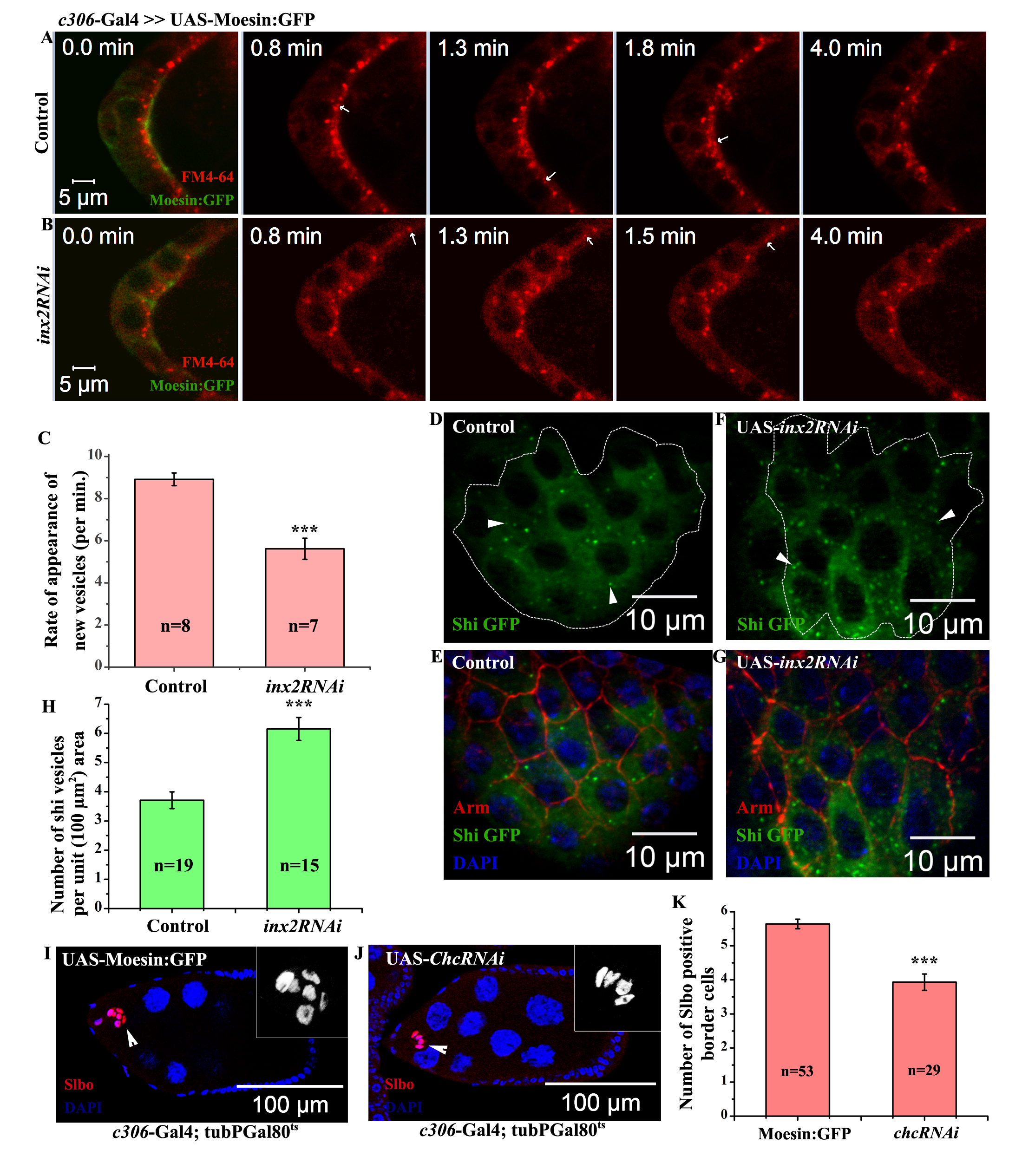

Supplement: S4 Fig — (A, B): Snapshot of time-lapse imaging of follicle cells of stage 8 egg chambers labeled with lipophilic dye FM4-64 (Red). Time interval is indicated in minutes (min). 0 min is the merged image of the Moesin:GFP and FM4-64 for the indicated genotypes. White arrows mark newly formed vesicle at the apical end. (C): Histogram compares the rate of appearance of vesicles per minute for genotypes indicated in (A) and (B). (D-G): Overexpression of UAS-ShiWT:GFP by c306-Gal4 driver in wild type (D, E) and Inx2-depleted (F, G) follicle cells. Follicle cell overexpressing ShiWT:GFP are outlined in white. GFP (Green) (D, F) and anti-Armadillo (Red) (E, G). Arrowheads mark the Shibire:GFP puncta. Note higher number of Shibire:GFP puncta in Inx2-depleted follicle cells compared to the control. (H). Quantification of Shibire:GFP puncta for genotypes represented in (D) and (F). (I, J) Stage 9–10 egg chamber of indicated genotype stained with anti-Slbo (Red) and DAPI (Blue). Arrowheads mark BC cluster and inset shows Slbo staining of the border cell cluster. (K) Histogram depicting the comparison of Slbo positive cells for genotypes represented in (I) and (J). ‘n’ indicates number of egg chambers analyzed. Error bar represents Standard Error of Mean. *** represents p-value <0.001. (TIF) [file pgen.1006542.s004.tif]

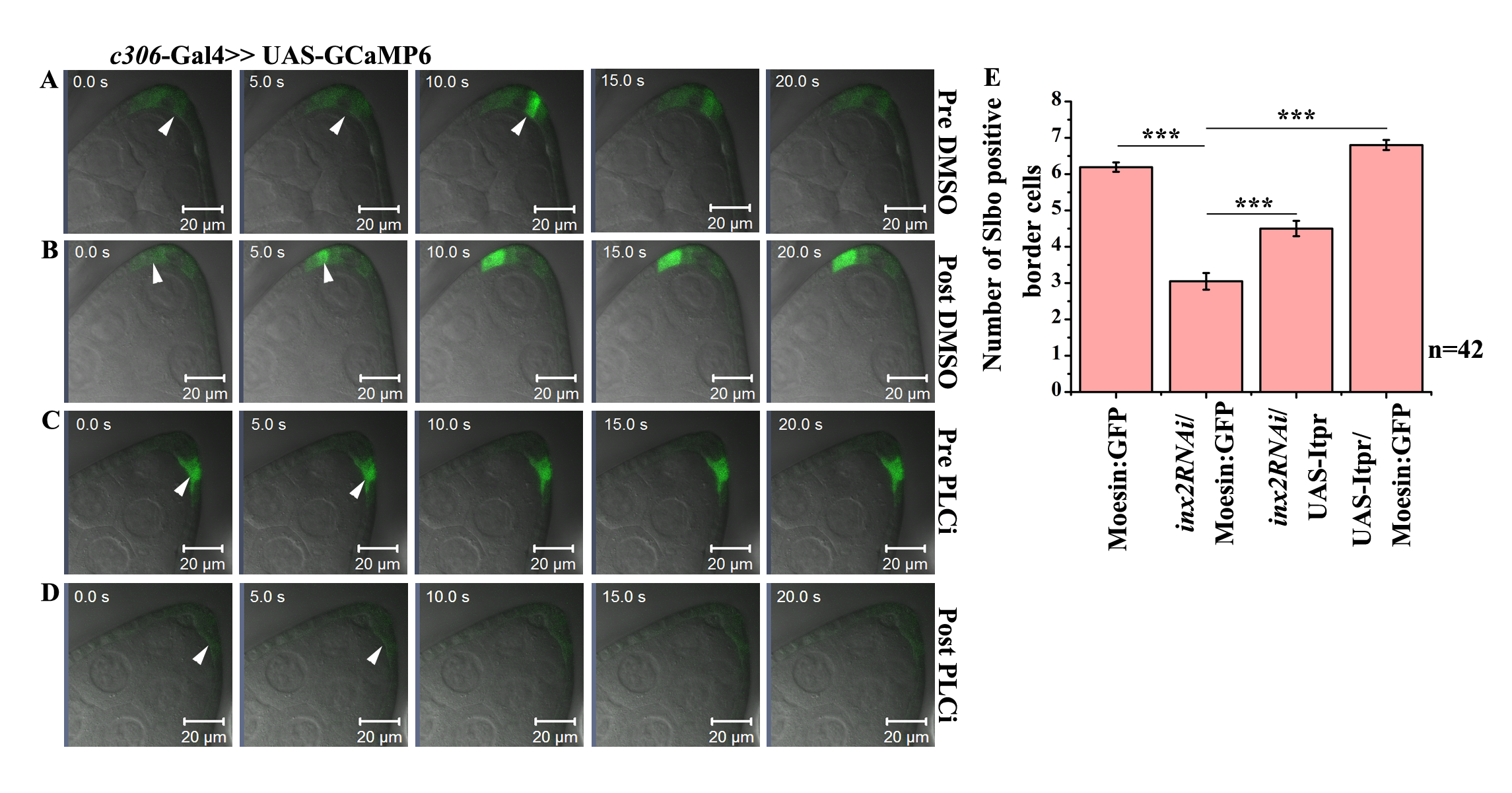

Supplement: S5 Fig — (A-D): Snapshots of Ca2+ flux in the egg chambers of indicated genotype under various conditions. Time is denoted in seconds. (A, B): pre- and post-DMSO treatment. (C, D): pre- and post-PLC inhibitor (PLCi) treatment. White arrowheads mark Ca2+ flux. The observed flux in the follicle cells is due to the release of intracellular Ca2+. (E): Histogram representing number of Slbo positive border cells in the indicated UAS lines driven by c306-Gal4. Note the rescue in border cell fate in Inx2-depleted clusters overexpressing UAS-Itpr. ‘n’ indicates number of egg chambers analyzed. Error bar represents Standard Error of Mean. *** represents p-value <0.001. (TIF) [file pgen.1006542.s005.tif]

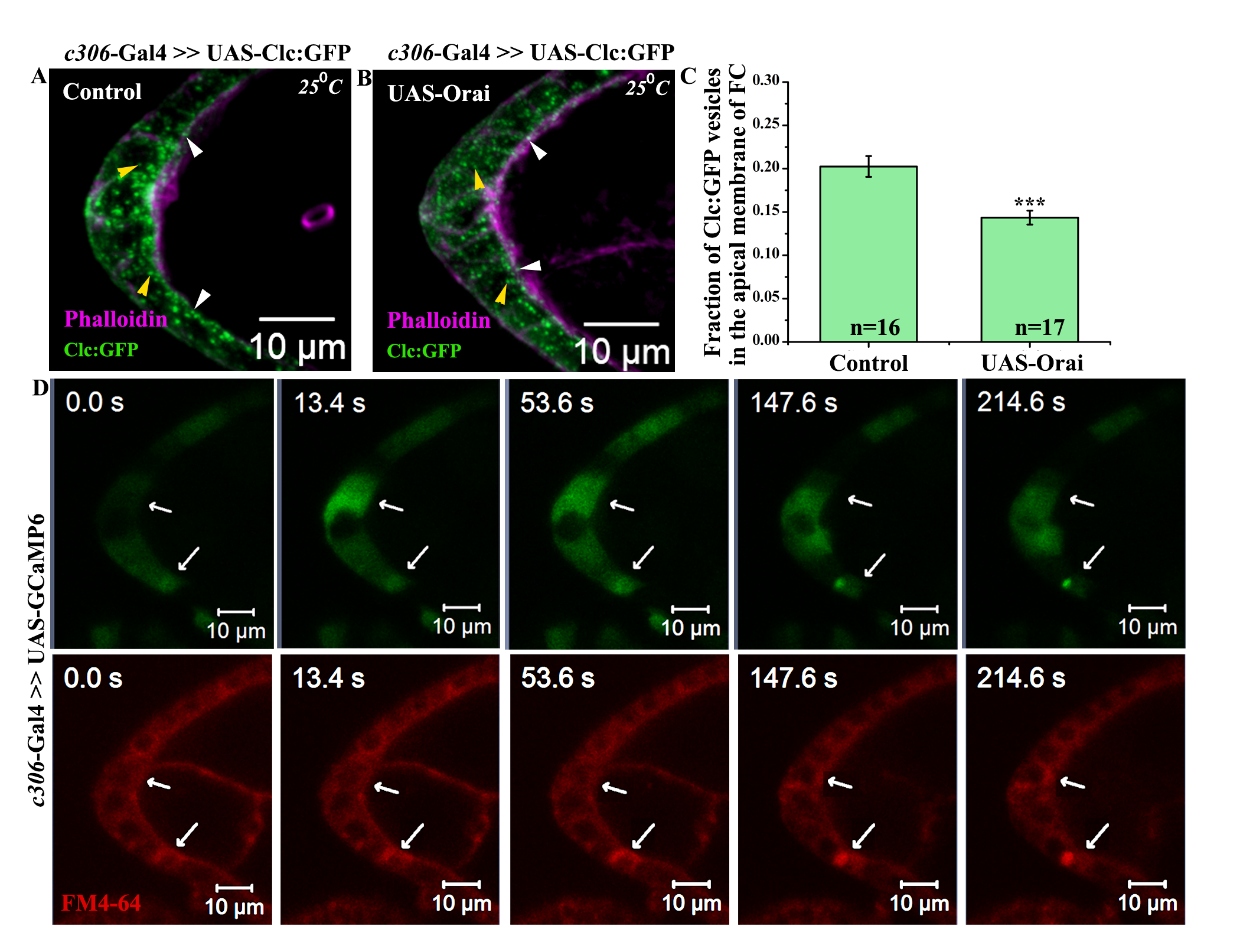

Supplement: S6 Fig — (A, B): Maximum intensity projection of egg chambers of indicated genotypes showing cellular distribution of Clathrin light chain GFP (Clc:GFP) vesicles in the anterior follicle cells. Rhodamine Phalloidin (Magenta) marks the outline of the egg chamber. White arrowheads indicate vesicles localized to apical membrane of follicle cells. Yellow arrowheads mark cytoplasmic vesicles. (C): Histogram compares apical fraction of Clc-GFP vesicles in the control and UAS-Orai over expressing follicle cells. (D): Snapshot of time lapse imaging of calcium flux in the presence of FM4-64 dye. Arrows mark the follicle cells with active calcium flux and it correlates with FM4-64 dye internalization. ‘n’ indicates number of egg chamber analyzed. Error bar represents Standard Error of Mean. *** represents p-value <0.001. (TIF) [file pgen.1006542.s006.tif]

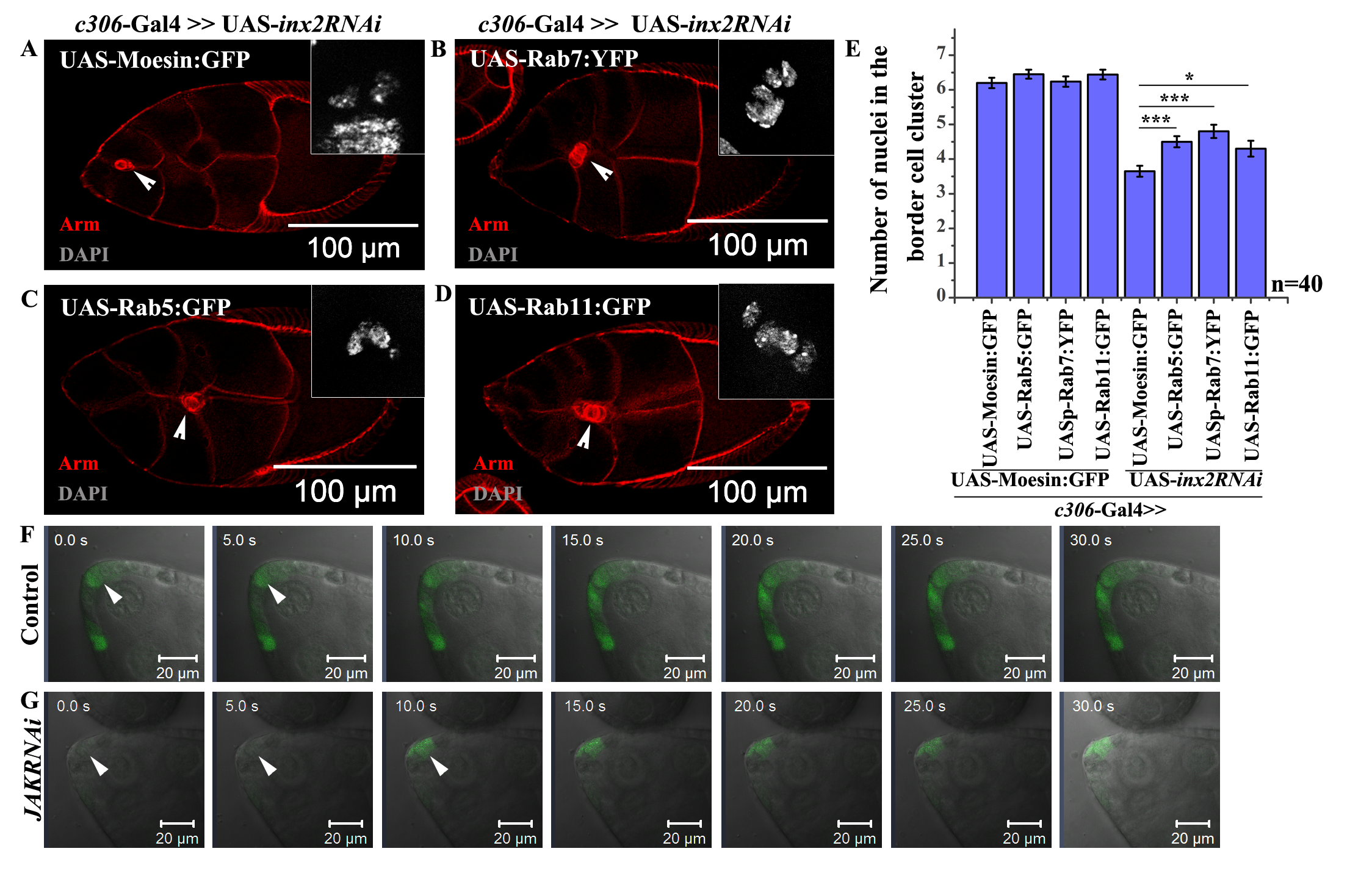

Supplement: S7 Fig — (A-D) Stage 9–10 egg chamber of indicated genotypes stained with anti-Armadillo (Red). Arrowheads mark border cell cluster and inset shows DAPI staining of border cell cluster. (E): Histogram showing number of nuclei in border cell clusters in the indicated genotypes. Note the rescue in BC number when endocytosis regulators are overexpressed. (F, G): Snapshots of time lapse imaging of calcium flux in control (c306-Gal4; UAS-GCaMP6) and Janus Kinase (JAK) RNAi (c306-Gal4; UAS-GCaMP6/ UAS-JAKRNAi) follicle cells. Arrowheads mark the calcium flux. No appreciable difference in the calcium flux was observed in follicle cells overexpressing JAKRNAi. ‘n’ indicates number of egg chamber analyzed. Error bar represents Standard Error of Mean. *** represents p-value <0.001, * represent p-value <0.05. (TIF) [file pgen.1006542.s007.tif]
